# CARM1 regulates tubulin autoregulation through PI3KC2α R175 methylation

**DOI:** 10.1186/s12964-025-02124-z

**Published:** 2025-03-05

**Authors:** Yena Cho, Jee Won Hwang, Mark T. Bedford, Dae-Geun Song, Su-Nam Kim, Yong Kee Kim

**Affiliations:** 1 Muscle Physiome Research Center and Research Institute of Pharmaceutical Sciences, Sookmyung Women’s University, Seoul, 04310 Republic of Korea; 2 College of Pharmacy, Sookmyung Women’s University, Seoul, 04310 Republic of Korea; 3 Department of Epigenetics and Molecular Carcinogenesis, The University of Texas MD Anderson Cancer Center, Houston, TX 77030 USA; 4 Natural Products Research Institute, KIST Gangneung, Gangneung, 25451 Republic of Korea; 5 Division of Natural Product Applied Science, University of Science and Technology KIST School, Seoul, 02792 Republic of Korea

**Keywords:** CARM1, PI3KC2α, Tubulin, Autoregulation, Mitosis

## Abstract

Tubulin is crucial in several cellular processes, including intracellular organization, organelle transport, motility, and chromosome segregation. Intracellular tubulin concentration is tightly regulated by an autoregulation mechanism, in which excess free tubulin promotes tubulin mRNA degradation. However, the details of how changes in free tubulin levels initiate this autoregulation remain unclear. In this study, we identified coactivator-associated arginine methyltransferase 1 (CARM1)-phosphatidylinositol 3-kinase class 2α (PI3KC2α) axis as a novel regulator of tubulin autoregulation. CARM1 stabilizes PI3KC2α by methylating its R175 residue. Once PI3KC2α is not methylated, it becomes unstable, leading to decreased cellular levels. Loss of PI3KC2α results in the release of tetratricopeptide repeat domain 5 (TTC5), which initiates tubulin autoregulation. Thus, PI3KC2α, along with its CARM1-mediated arginine methylation, regulates the initiation of tubulin autoregulation. Additionally, disruption of the CARM1-PI3KC2α axis decreases intracellular tubulin levels, leading to a synergistic increase in the cytotoxicity of microtubule-targeting agents (MTAs). Taken together, our study demonstrates that the CARM1-PI3KC2α axis is a key regulator of TTC5-mediated tubulin autoregulation and that disrupting this axis enhances the anti-cancer activity of MTAs.

**Supplementary Information:**

The online version contains supplementary material available at 10.1186/s12964-025-02124-z.

## Introduction

Microtubules, composed of repeating α- and β-tubulin subunits, are essential structural components of the cytoskeleton [1, 2]. Their dynamic properties arise from the continuous polymerization and depolymerization of tubulin subunits, processes tightly regulated by multiple factors, including the intracellular abundance of soluble tubulin, microtubule-associated proteins, and post-translational modifications (PTMs) of tubulin [3–5]. To maintain microtubule homeostasis, cells employ tubulin autoregulation, a translation-dependent mechanism that selectively degrades tubulin-encoding mRNAs in response to excess free tubulin, thereby preventing its overproduction [6, 7]. A key regulator of tubulin autoregulation is tetratricopeptide repeat domain 5 (TTC5), which recognizes a conserved motif in nascent tubulin polypeptides emerging from ribosomes [8]. TTC5 then recruits the adaptor protein SCAPER, the substrate-recruitment subunit CNOT11, and the CCR4–NOT deadenylase complex, leading to tubulin mRNA degradation and fine-tuning tubulin levels [8, 9]. Despite the established role of TTC5 in this process, the molecular mechanisms by which cells sense and respond to fluctuations in free tubulin remain incompletely understood, necessitating the identification of additional regulatory factors.

**Fig. 1:**
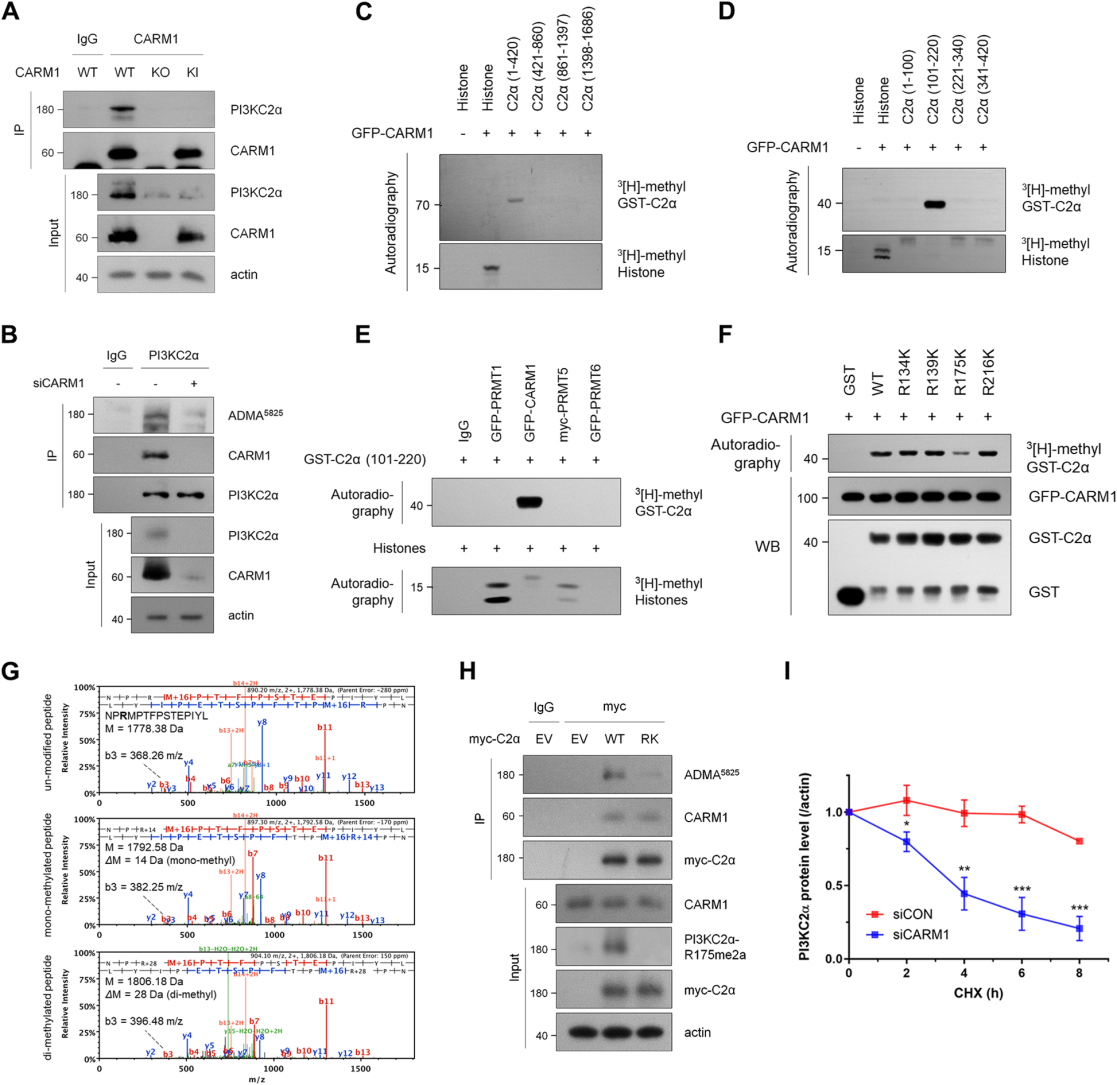
CARM1 methylates and stabilizes PI3KC2α at R175 residue. **A** and **B** The interaction between PI3KC2α and CARM1 as well as the methylation of PI3KC2α by CARM1 were confirmed by performing immunoprecipitation (IP) of anti-CARM1 antibody from CARM1 wild-type (WT), knockout (KO), or CARM1 R169A knock-in (KI) MEFs (**A**). CARM1-PI3KC2α interaction was detected by immunoblotting after immunoprecipitation of PI3KC2α in CARM1-KD cells, and the methylation level of PI3KC2α was measured using an anti-ADMA^5825^ antibody (**B**). **C** to **F** CARM1 methylates the R175 site of PI3KC2α. An in vitro methylation assay was performed using GST-fusion peptides from PI3KC2α as CARM1 substrates (**C** and **D**). GST-PI3KC2α (101–220) was methylated in vitro by CARM1 but not by PRMT1, PRMT5, or PRMT6 (**E**). GST-PI3KC2α RK mutants (residues 101–220; R134K, R139K, R175K, and R216K represent the only arginine residues present between 101–220) were methylated in vitro by CARM1, but the methylation signal was reduced only in R175K (**F**). **G** MS/MS spectra of R175-containing peptides. Compared with unmodified peptides, monomethylated and dimethylated peptides had masses of 14 and 28 Da, respectively. The mass difference of b3 ions, which are indicative of the third amino acid residue, R175, also showed the same mass difference as the parent mass. **H** After myc-PI3KC2α WT or R175K mutant construct was transfected into MCF7 cells, myc-PI3KC2α was immunoprecipitated. **I** CARM1 stabilizes the PI3KC2α protein by methylating the R175 residue. Cycloheximide (CHX, 50 μg/ml) was added to MCF7 cells transfected with control or CARM1 siRNA for 72 h. Error bar represents the standard deviation of three independent experiments

Recent study suggests a potential link between phosphatidylinositol 3-kinase C2α (PI3KC2α) and microtubule regulation [10]. Unlike Class I PI3Ks, which mediate growth factor signaling, Class II PI3Ks—including PI3KC2α, PI3KC2β, and PI3KC2γ—primarily function in membrane trafficking, endocytosis, and cytoskeletal organization by catalyzing the synthesis of phosphoinositides such as phosphatidylinositol 3-phosphate (PI(3)P) and phosphatidylinositol 3,4-bisphosphate (PI(3,4)P_2_) [11–14]. Among these isoforms, PI3KC2α is indispensable for embryonic development in mice [12], and loss of its catalytic activity is associated with defects in endocytosis [15, 16], angiogenesis and endothelial cell function [17–19], and primary cilia signaling [12]. Furthermore, homozygous loss-of-function mutations in *PIK3C2A* have been linked to developmental disorders characterized by short stature, craniofacial abnormalities, cataracts, skeletal defects, and neurological impairments [20]. Notably, PI3KC2α localizes to spindle poles and ensures proper chromosome segregation during mitosis by stabilizing microtubules [10]. It implies that PI3KC2α may contribute to tubulin homeostasis. Meanwhile, an analysis of the PhosphoSite database (www.phosphosite.org) revealed that PI3KC2α undergoes arginine methylation, a PTM catalyzed by protein arginine methyltransferases (PRMTs) [21, 22]. Indeed, we identified a regulatory link between CARM1 and PI3KC2α by observing a marked reduction in PI3KC2α expression in coactivator-associated arginine methyltransferase 1 (CARM1)-deficient cells (Fig. 1A). Given that PRMTs, including CARM1, play crucial roles in cellular homeostasis functions [23–25], we hypothesized that CARM1-mediated methylation of PI3KC2α might influence tubulin autoregulation.

In this study, we investigated the CARM1–PI3KC2α axis in tubulin autoregulation. By elucidating this pathway, we aimed to determine how cells maintain tubulin homeostasis and whether PI3KC2α serves as a key mediator in this process. Our findings provide novel insights into microtubule regulation and highlight potential therapeutic targets for disorders associated with cytoskeletal dysregulation.

## Results

### CARM1 methylates and stabilizes PI3KC2α at R175 residue

Although PI3KC2α is ubiquitously expressed across all tissues [26], we found that its expression depended on intracellular CARM1 levels and activity (Fig. 1A, B). Moreover, CARM1 interacted with and methylated PI3KC2α (Fig. 1A, B, S1A). These findings suggest that PI3KC2α serves as a novel substrate for CARM1 and that its intracellular function can be regulated by CARM1. An in vitro methylation assay identified R175 as the specific methylation site on PI3KC2α, which was validated using deletion mutants (Fig. 1C-E), point mutants (Fig. 1F), and mass spectrometry (Fig. 1G). R175 methylation was further confirmed in vivo, as the methylation signal, clearly detected by a PI3KC2α R175me2a-specific antibody, was markedly decreased in cells expressing the R175K mutant (Fig. 1H, S1B). We also observed that PI3KC2α protein levels markedly reduced in two other cell lines, MCF7 and HeLa after knocking down CARM1 with specific siRNA (Fig. S1C). Interestingly, the *PI3KC2α* mRNA expression was not affected by CARM1 (Fig. S1D), indicating that CARM1 contributes to PI3KC2α protein stability, rather than affecting its transcription. Indeed, PI3KC2α levels in CARM1-knockdown (KD) cells (Fig. 1I, S1E) and cells expressing the R175K mutant (Fig. S1F) more rapidly reduced in the presence of cycloheximide. Moreover, PI3KC2α ubiquitination was enhanced by knocking down CARM1 or overexpressing the R175K mutant, with further increases observed upon treatment with MG132 (Fig. S1G, S1H).

### CARM1–PI3KC2α axis determines intracellular tubulin levels

**Fig. 2:**
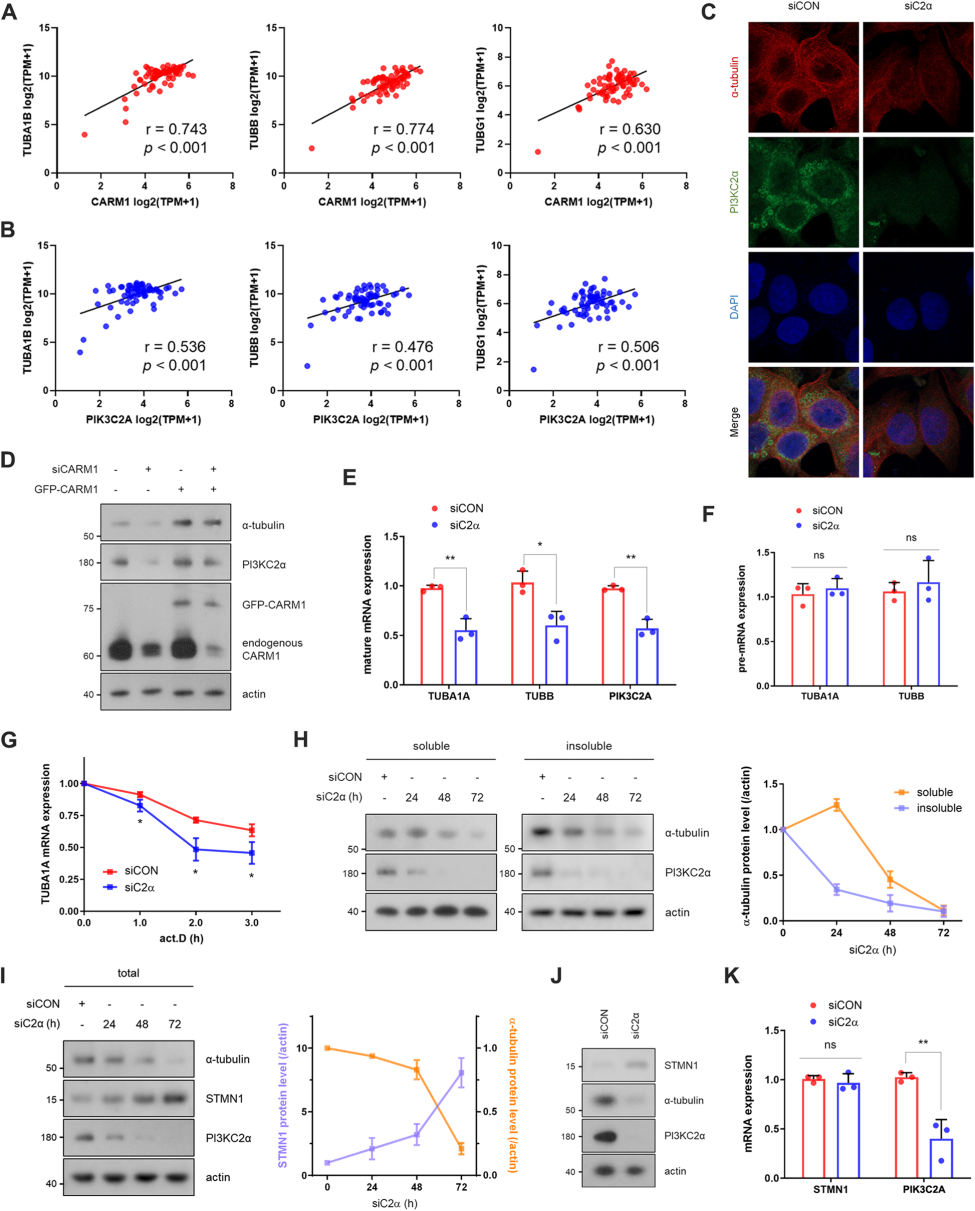
CARM1–PI3KC2α axis determines intracellular tubulin levels. **A** and **B** Correlation analysis of tubulin versus *CARM1* (**A**) or *PIK3C2A* (**B**) using Pearson correlation in 67 breast cancer cell lines, derived from DepMap Expression Public 24Q2 data. Each dot represents a cell line and the black line indicates a linear regression line. **C** Representative confocal images showing α-tubulin (red) and PI3KC2α (green) levels. DAPI (blue) was used to stain cell nuclei. **D** After CARM1 was rescued with a GFP-CARM1 plasmid, the cell lysates were analyzed by immunoblotting to detect the indicated proteins. **E** and **F** The levels of mature mRNA (**E**) and pre-mRNA (**F**) of each tubulin (*TUBA1A* or *TUBB*) were measured in PI3KC2α-knockdown MCF7 cells. The error bar indicates the standard deviation (*n* = 3). **G** After transfection with PI3KC2α siRNA for 60 h, MCF7 cells were treated with actinomycin D (act. D) for 1, 2, and 3 h. *TUBA1A* mRNA levels at each time point are presented as relative values to those of cells not treated. The error bar represents the standard deviation (*n* = 3). **H** and **I** After transfection with PI3KC2α siRNA for different durations (24, 48, and 72 h), soluble and insoluble fractions were immunoblotted to measure α-tubulin levels (**H**), and total α-tubulin and STMN1 levels in total lysates were measured (**I**). The level of each protein in the graphs was normalized to that of the loading control (actin). **J** and **K** The STMN1 protein (**J**) and mRNA (**K**) levels were measured in PI3KC2α-knockdown cells

Given that PI3KC2α stabilizes the mitotic spindle during mitosis [10], we aimed to investigate whether the CARM1–PI3KC2α axis affects microtubule regulation. The expression of *CARM1* or *PIK3C2A* was correlated with tubulin expression (*TUBA1A*, *TUBA1B*, *TUBA1C*, *TUBB*, *TUBB2A*, *TUBB3*, *TUBB4B*, *TUBB6*, and *TUBG1*) in 67 breast cancer cell lines from the DepMap database (Fig. 2A, B, S2A). We found that depleting PI3KC2α reduced α-tubulin levels in immunostaining results (Fig. 2C). Other tubulin isoforms (α-, β-, and γ-) were also remarkably reduced in KD cells (Fig. S2B). CARM1-KD or -knock out (KO) cells also exhibited decreased α-tubulin expression, accompanied by a concurrent reduction in PI3KC2α (Fig. 2D, S2C, S2D). Meanwhile, restoration of CARM1 in CARM1-KD or -KO cells rescued PI3KC2α and α-tubulin levels (Fig. 2D, S2D). These findings suggest that the CARM1–PI3KC2α axis is likely critical in determining intracellular tubulin levels.

Intracellular tubulin levels are tightly regulated through autoregulation, a feedback loop that impacts mRNA stability [6]. Thus, to investigate whether changes in tubulin levels introduced by the CARM1–PI3KC2α axis are caused by tubulin autoregulation, we analyzed tubulin mature mRNA and pre-mRNA. PI3KC2α KD decreased *TUBA1A* and *TUBB* mature mRNA levels without impacting their pre-mRNA levels (Fig. 2E, F); similar results were observed when CARM1 was knocked down (Fig. S2E, S2F). Additionally, the half-life of *TUBA1A* mRNA was markedly shorter in the PI3KC2α-KD group than in the control group (Fig. 2G, S2G), indicating that PI3KC2α modulates tubulin mRNA stability rather than transcription. Considering that PI3KC2α predominantly existed in the cytoplasm (Fig. S2H), we excluded the possibility of it regulating splicing steps. Moreover, PI3KC2α does not affect S6 phosphorylation—involved in the translation process (Fig. S2I, S2J), leading us to conclude that PI3KC2α also does not likely impact translation. Since excess soluble tubulins initiate tubulin autoregulation, we next investigated whether depletion of PI3KC2α altered the ratio of soluble tubulin to insoluble microtubules. PI3KC2α KD initially increased the levels of soluble free tubulin while decreasing those of insoluble microtubules, subsequently leading to a marked decrease in both soluble and insoluble tubulin levels (Fig. 2H). Furthermore, the intracellular levels of tubulin in PI3KC2α- or CARM1-KD cells were inversely correlated with the levels of the microtubule destabilizer STMN1 (Fig. 2I, J, S2K). Specifically, PI3KC2α or CARM1 KD increased STMN1 protein levels without affecting mRNA levels (Fig. 2J, K, S2K, S2L).

### PI3KC2α regulates tubulin autoregulation by sequestering TTC5

**Fig. 3:**
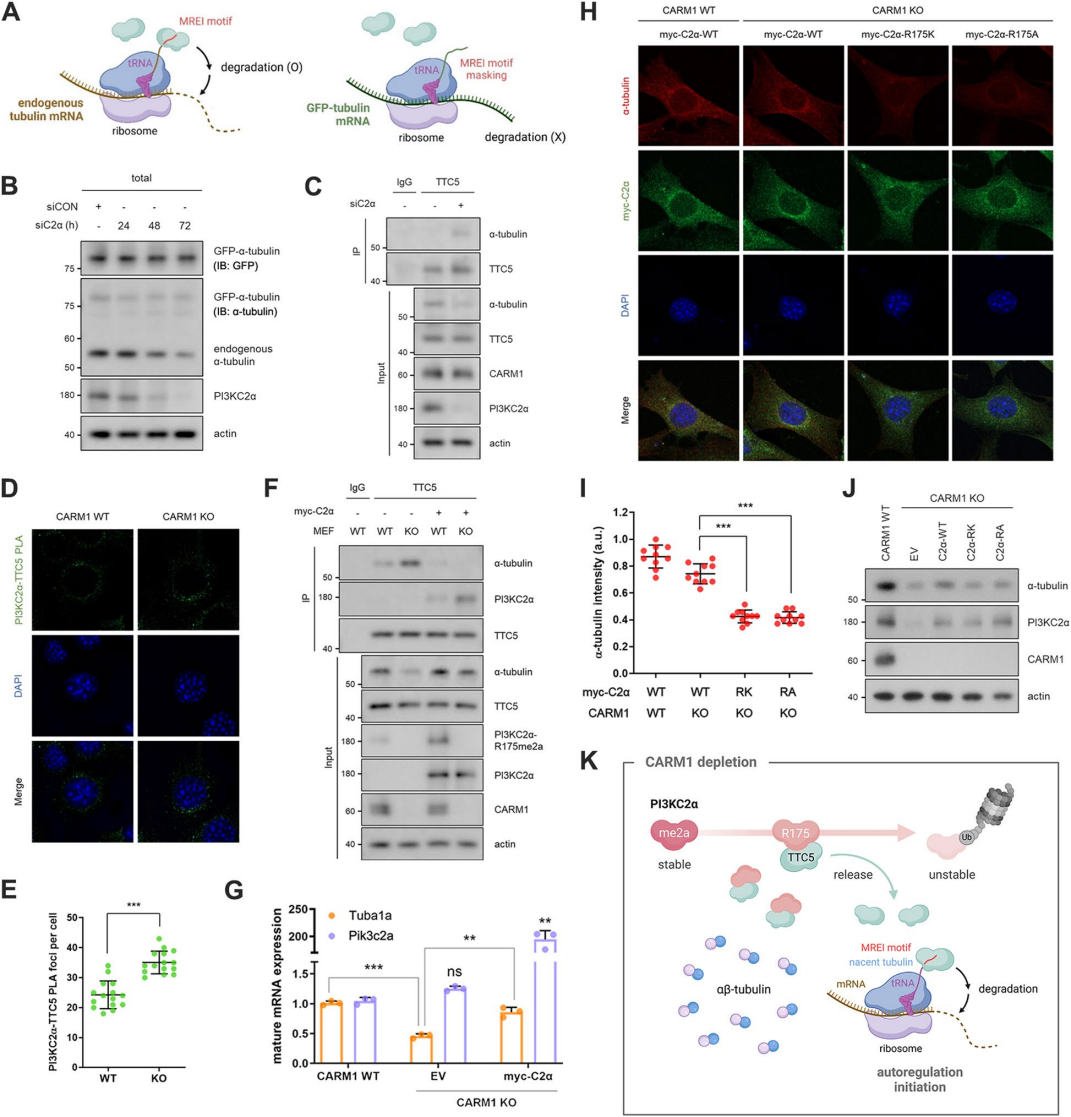
PI3KC2α regulates tubulin autoregulation by sequestering TTC5. **A** Schematic images of TTC5-mediated tubulin autoregulation. **B** HeLa cells stably expressing GFP-α-tubulin were transfected with PI3KC2α siRNA for 24, 48, or 72 h. Total lysates were analyzed by immunoblotting analysis. **C** Physical interactions of α-tubulin with TTC5 were measured using co-IP experiments after transfecting the cells with PI3KC2α siRNA. **D** and **E** Interaction between PI3KC2α and TTC5 recognized by a PLA probe. After ligation and amplification, PLA signals (green dots) were counted. The nuclei were stained with DAPI (blue). Data are shown as mean ± standard deviation (*n* = 15). **F** and **G** CARM1 WT or KO MEFs were transfected with the myc-PI3KC2α vector. The cell lysates were immunoprecipitated with an anti-TTC5 antibody. The input and immunoprecipitates were immunoblotted to detect the indicated proteins (**F**). Mature *Tuba1a* and *Pik3c2a* mRNA levels in each group were measured (**G**). **H** to **J** Confocal images (**H** and **I**) and immunoblot analysis (**J**) showing α-tubulin (red) levels in CARM1 KO MEF cells transfected with WT, R175K-, or R175A-mutant of myc-PI3KC2α (green). **K** Schematic representation of CARM1–PI3KC2α axis in tubulin autoregulation pathway. Unmethylated PI3KC2α stabilizes free tubulin and disrupts tubulin autoregulation by sequestering TTC5. When PI3KC2α becomes unstable and degrades, TTC5 is released and triggers tubulin autoregulation

Tubulin autoregulation is initiated when TTC5 recognizes the MREI sequence in the nascent peptide of tubulin in ribosomes [7, 8]. To investigate if the CARM1–PI3KC2α axis balances intracellular tubulin levels through the TTC5-mediated autoregulation pathway, we used GFP-tubulin expressing cells with the N-terminal MREI sequence masked by GFP (Fig. 3A). Although PI3KC2α or CARM1 KD decreased endogenous tubulin, it did not impact GFP-tubulin (Fig. 3B, S3A). PI3KC2α or CARM1 KD also substantially increased the interaction between TTC5 and α-tubulin (Fig. 3C, S3B), indicating the involvement of CARM1-PI3KC2α axis in tubulin autoregulation. We then investigated whether CARM1-mediated methylation of PI3KC2α affected its function as a mediator of tubulin autoregulation. CARM1 KD decreased the R175 methylation level of PI3KC2α and increased its interaction with TTC5 (Fig. S3C), indicating that the unmethylated form of PI3KC2α was more likely to interact with TTC5. Moreover, in CARM1-KO cells, PI3KC2α interacted better with TTC5 despite its decreased levels than in CARM1-WT cells (Fig. 3D, E). The overexpression of PI3KC2α WT also increased the interaction between TTC5 and PI3KC2α and restored α-tubulin protein and mRNA levels in CARM1-KO cells (Fig. 3F-J, S3D), whereas R175K or R175A mutant did not affect α-tubulin levels (Fig. 3H-J). These findings highlight that intact R175 form of PI3KC2α is essential to maintain a certain amount of tubulin. Taken together, unmethylated PI3KC2α preferentially interacts with and sequesters TTC5, preventing its recognition of nascent tubulin peptides and temporarily suppressing tubulin autoregulation. However, as unmethylated PI3KC2α is unstable and rapidly degrades (see Fig. 1), TTC5 is released, enabling tubulin autoregulation to proceed (Fig. 3K).

### Disruption of the CARM1–PI3KC2α axis enhances the cytotoxic effects of microtubule-targeting agents

**Fig. 4:**
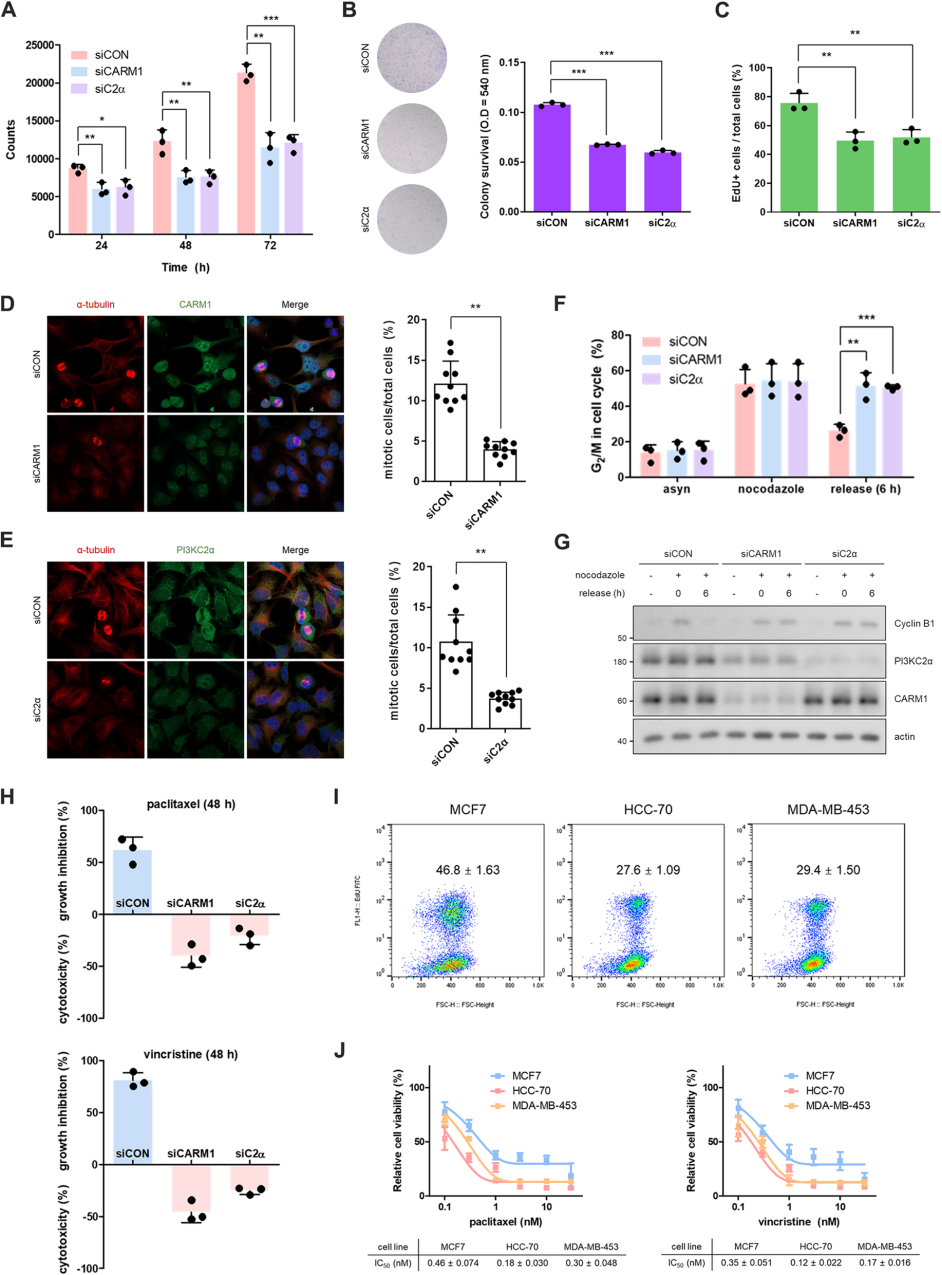
Disruption of the CARM1–PI3KC2α axis enhances the cytotoxic effects of microtubule-targeting agents. **A** and **B** MCF7 cells were transfected with control, CARM1, or PI3KC2α siRNA for 72 h and then replated with fresh media. A Coulter counter was used for cell counting after 24, 48, and 72 h of incubation (**A**). Colonies were stained with crystal violet, and the absorbance was measured at 540 nm after 120 h of incubation (**B**). The data are presented as the means ± standard deviations (*n* = 3). **C** After CARM1- or PI3KC2α-knockdown cells were incubated with 20 μM EdU for 3 h, the EdU-positive cells were counted using flow cytometry. The data are presented as the means ± standard deviations (*n* = 3). **D** and **E** After HeLa cells were transfected with siRNAs against PI3KC2α (**D**) or CARM1 (**E**) for 72 h, the cells were costained with antibodies against CARM1 (green) or PI3KC2α (green) and α-tubulin (red). The nuclei were visualized via DAPI (blue) staining. Mitotic cells were counted and are expressed as a relative percentage. The data are presented as the means ± standard deviations (*n* = 10). **F** and **G** After synchronizing CARM1- or PI3KC2α-knockdown cells in prometaphase with nocodazole, the cells were released by incubating with fresh media for 6 h. The cell cycle was analyzed using flow cytometry (**F**) and immunoblotting (**G**). **H** MCF7 cells were transfected with siRNA targeting CARM1 or PI3KC2α. The cells were then replated with fresh media and treated with either 3 nM paclitaxel or vincristine. Cell counting was performed after 48 h of incubation. The results are presented as the percentage of cells in each group after paclitaxel or vincristine treatment. A positive value indicated growth inhibition (%), while a negative value indicated cytotoxicity (%). The data are expressed as the means ± standard deviations (*n* = 3). **I** Cell proliferation rates of MCF7, HCC-70, and MDA-MB-453 cells were assessed through an EdU incorporation assay. **J** The growth inhibitory effects of paclitaxel or vincristine on MCF7, HCC-70, and MDA-MB-453 cells were measured, and the IC_50_ values were determined using GraphPad Prism software. The data are presented as the means ± standard deviations (*n* = 3)

As the CARM1–PI3KC2α axis regulates microtubule dynamics, we examined the effects of depleting CARM1 or PI3KC2α on cancer cell proliferation. The knockdown of CARM1 or PI3KC2α in breast cancer MCF7 cells significantly inhibited cell proliferation (Fig. 4A), cell survival (Fig. 4B), and DNA synthesis (Fig. 4C, S4A, S4B). Furthermore, the population of mitotic cells in the CARM1- or PI3KC2α-KD group was lower than that in the control group (siCON = 31.3%, siPI3KC2α = 9.0%, and siCARM1 = 8.1%; Fig. 4D, E). Hence, the depletion of CARM1 or PI3KC2α affected mitosis in terms of cell cycle progression. This effect on mitosis was assessed by arresting the cell cycle in the G_2_/M phase using nocodazole. After 72 h of CARM1 or PI3KC2α KD, the cells were synchronized in the G_2_/M phase via nocodazole treatment and subsequently released. After 6 h of release, most cells in the control group moved to G_1_ phase, while those in either CARM1 or PI3KC2α siRNA-treated groups were retained in the G_2_/M phase (Fig. 4F, S4C). Additionally, after release from nocodazole treatment, CARM1- or PI3KC2α-KD cells sustained Cyclin B1 levels, which is specific to the G_2_/M stage (Fig. 4G). Hence, disruption of the CARM1–PI3KC2α axis impeded mitotic progression and consequently induced growth retardation.

We also evaluated this effect in Rb-null cell line MDA-MB-468, which exhibits low tubulin levels due to activation of E2F-dependent transcription of STMN1 [27] (Fig. S4D). Knockdown of PI3KC2α interfered with microtubule formation, as indicated by a decrease in insoluble tubulin levels (Fig. S4E). Moreover, confocal images revealed that depletion of PI3KC2α resulted in mitotic failure, such as impaired spindle fiber formation, chromosome condensation, and chromosome segregation (Fig. S4F), ultimately leading to suppressed cell growth (Fig. S4G). Finally, we evaluated the effect of downregulating CARM1 or PI3KC2α on the sensitivity of MCF7 cells to microtubule-targeting agents (MTAs), namely paclitaxel and vincristine. Downregulating PI3KC2α or CARM1 inhibited cell proliferation and increased sensitivity to both MTAs (Fig. 4H, S4H). Additionally, we compared the cell growth and sensitivity of several human breast cancer cell lines to MTAs based on the level of PI3KC2α. HCC-70 and MDA-MB-453 cells exhibited lower levels of PI3KC2α and α-tubulin than MCF7 cells (Fig. S4I, S4J). Consistent with the siRNA treatment results, the growth rate of HCC-70 and MDA-MB-453 cells was slower (Fig. 4I), and the IC_50_ values of paclitaxel and vincristine were lower than in MCF7 cells (Fig. 4J). Hence, decreased PI3KC2α suppressed cell proliferation and enhanced sensitivity to paclitaxel and vincristine.

## Discussion

Tubulin autoregulation, proposed approximately 40 years ago, is a crucial mechanism for maintaining intracellular tubulin levels by promoting tubulin mRNA degradation when excess free tubulin is detected. Recent studies have identified TTC5 as a key factor in this process, initiating it by binding to nascent tubulin polypeptides and recruiting factors that degrade tubulin mRNA [8, 9]. Additionally, it has been reported that newly translated soluble αβ-tubulin dimers sequester TTC5, but free αβ-tubulin dimers released by microtubule destabilization reduce these interactions, thereby releasing TTC5 and promoting autoregulation [28]. However, the mechanism by which free αβ-tubulin dimers released from microtubule destabilization disrupt these interactions and release TTC5 remains unclear. In this context, our model of PI3KC2α-mediated TTC5 release provides a clearer explanation of how the TTC5-mediated tubulin autoregulation pathway is triggered. Specifically, during microtubule destabilization, TTC5 appears to be temporarily sequestered by unmethylated PI3KC2α, preventing premature activation of the tubulin autoregulation pathway. This sequestration likely persists until PI3KC2α undergoes proteolytic degradation, at which point TTC5 is released, initiating the degradation of tubulin mRNA. Our results also suggest that methylated PI3KC2α has reduced binding affinity for TTC5, leading to an increase in free TTC5. However, tubulin autoregulation is not immediately triggered under these conditions, possibly because methylated PI3KC2α promotes microtubule polymerization, thereby reducing the overall pool of free tubulin. As a result, newly translated αβ-tubulin dimers could sequester TTC5, preventing the activation of autoregulation.

Mutations in *PIK3C2A* have been linked to a genetic syndrome characterized by dysmorphic features, short stature, skeletal abnormalities, and cataracts associated with ciliary dysfunction [20]. Given the established role of PI3KC2α in spindle assembly [10] and early endosome formation [12, 29, 30], these phenotypes may, at least in part, result from disruptions in microtubule dynamics. Our findings further suggest that PI3KC2α is involved in tubulin autoregulation via CARM1-mediated R175 methylation, raising the possibility that *PIK3C2A* mutations impair tubulin homeostasis across multiple tissues, potentially contributing to these developmental defects. Further investigation into the interplay between CARM1 and PI3KC2α in regulating microtubule stability, intracellular trafficking, and cytoskeletal integrity may provide deeper insights into the molecular pathogenesis of these developmental defects.

Microtubules are highly dynamic structures that are constantly polymerized and depolymerized, a property that is crucial for their role in mitotic division [31]. Imbalanced microtubule dynamics can lead to several mitotic defects, resulting in failed cell division [32]. We have shown that disrupting the CARM1–PI3KC2α axis substantially impedes cell cycle progression and impacts microtubule dynamics by arresting in the mitotic stage, effectively inhibiting cancer cell proliferation. Indeed, certain chemotherapeutic drugs, such as paclitaxel and vincristine, target microtubules and disrupt their dynamics, inhibiting cancer cell division. Although these MTAs are widely used to treat breast cancer, including triple-negative breast cancer, they elicit serious adverse effects that limit their effectiveness [33–35]. Hence, research has shifted to identify selective biomarkers or develop combination therapies based on synthetic lethality. A recent report has suggested that intracellular tubulin levels are crucial for determining susceptibility to MTAs, as evidenced by MTAs induce synthetic lethality in cancer cells with dysregulated microtubule dynamics [27]. Consistent with this observation, downregulating CARM1 or PI3KC2α significantly increases sensitivity to paclitaxel and vincristine. Additionally, the susceptibility of human breast cancer cell lines (MCF7, HCC-70, and MDA-MB-453) to MTAs depends on tubulin and PI3KC2α levels. These findings suggest that CARM1 or PI3KC2α could serve as biomarkers or therapeutic targets for synthetic lethality-based MTA chemotherapy. Further research into the regulatory mechanisms underlying the CARM1-PI3KC2α axis in microtubule dynamics may provide novel therapeutic strategies for improving cancer treatment efficacy while minimizing adverse effects.

## Methods

### Chemicals, plasmids and antibodies

Actinomycin D, cycloheximide, paclitaxel, rapamycin, and vincristine were purchased from Sigma‒Aldrich (St. Louis, MO, USA). MS-grade LC‒MS/MS solvents, including 0.1% formic acid in acetonitrile and 0.1% formic acid in water, were purchased from Fisher Scientific (Pittsburgh, PA, USA). Sequencing grade trypsin and chymotrypsin were obtained from Promega (Madison, WI, USA). Dithiothreitol, iodoacetamide, ammonium bicarbonate, and all other reagents were obtained from Sigma‒Aldrich.

GFP-CARM1 plasmid was provided by Dr. Mark T. Bedford (University of Texas MD Anderson Cancer Center), and myc-PI3KC2α was provided by Dr. Emily Kim Malmberg (Biotech Research and Innovation Centre, University of Copenhagen). To generate deletion mutants of PI3KC2α, the fragmented polymerase chain reaction (PCR) products obtained from the full-length myc-PI3KC2α were cloned and inserted into the pGEX-6p-1 vector.

The following antibodies were used for Immunoblotting and immunoprecipitation: β-actin (Santa Cruz Biotechnology, Dallas, TX, USA, sc-47778), CARM1 (Bethyl Laboratories, Montgomery, TX, USA, A300-421A), Cyclin B1 (Cell Signaling Technology, Danvers, MA, USA, #12231), GFP (Santa Cruz Biotechnology, sc-9996), GST (Santa Cruz Biotechnology, sc-138), HA (Cell Signaling Technology, #3724), Histone H3 (Cell Signaling Technology, #9715), c-Myc (Santa Cruz Biotechnology, sc-40), PI3KC2α (BD Biosciences, Franklin Lakes, NJ, USA, #611046, Proteintech, Rosemont, IL, USA, 22028–1-AP, and Santa Cruz Biotechnology, sc-365290), Rb (Cell Signaling Technology, #9309), S6 (Cell Signaling Technology, #2217), phospho-S6 (Cell Signaling Technology, #4858), STMN1 (Cell Signaling Technology, #3352), TTC5 (Proteintech, 26112–1-AP), α-tubulin (Santa Cruz Biotechnology, sc-5286 and Cell Signaling Technology, #2144), β-tubulin (Sigma‒Aldrich, T8660), and γ-tubulin (Bethyl Laboratories, A302-631A). The ADMA^5825^ antibody, which recognizes CARM1 substrates, was obtained from Dr. Mark T. Bedford. The PI3KC2α-R175me2a antibody (AbClon, Seoul, Republic of Korea, AC230110-161) was generated in rabbits using asymmetrically dimethylated peptide (NH_2_-NGFNPR(me2a)MPTFP-COOH). HRP-conjugated secondary antibodies were purchased from Jackson ImmunoResearch Laboratories (West Grove, PA, USA, 111–035-003 and 115–035-003).

### Cell culture and transfection

MCF7, HeLa, 293 T, and MDA-MB-468 cells were obtained from the American Type Culture Collection (Manassas, VA, USA), whereas HCC-70 and MDA-MB-453 cells were purchased from the Korean Cell Line Bank (Seoul, Republic of Korea). MEFs (CARM1 wild-type, knockout and R169A knock-in) were obtained from Dr. Mark T. Bedford, and GFP-α-tubulin stable HeLa cells were donated by Dr. Chang-Young Jang (Sookmyung Women’s University, Seoul, Republic of Korea). MEF, 293 T, HeLa, and MCF7 cells were grown in DMEM (HyClone Laboratories, Logan, UT, USA), and MDA-MB-468, HCC-70, and MDA-MB-453 cells were cultured in RPMI-1640 (HyClone Laboratories). Both cell culture media were supplemented with 10% fetal bovine serum (HyClone Laboratories) and 100 units/ml penicillin/streptomycin (HyClone Laboratories). The cells were maintained at 37 °C in a humidified cell culture incubator containing 5% CO_2_. Furthermore, siRNAs were transfected into cells using TransIT-X2 (Mirus Bio, Madison, Wisconsin, USA), and DNA was transfected into cells using TransIT-2020 (Mirus Bio) in accordance with the manufacturer’s instructions.

### In vitro methylation assay

In vitro methylation assay was performed as described previously [36, 37]. PRMT1, PRMT4, PRMT5, or PRMT6 was purified from 293 T cells that were transfected with GFP- or myc-PRMT vector. After immunoprecipitation with anti-GFP or myc antibody, each purified protein was incubated with 50 μL of reaction buffer (20 mM Tris–HCl with pH 7.5, 150 mM NaCl, 2 mM EDTA, 1 mM PMSF, and 1 mM DTT) supplemented with 1 μg of recombinant histones (mixture of H2A, H2B, H3, and H4; New England Biolabs, Ipswich, MA) or 1 μg of GST-PI3KC2α and 1 μCi ^3^[H]-AdoMet (specific activity: 55–85 Ci/mmol; PerkinElmer, Waltham, MA) at 37 °C for 1 h. The reaction was terminated by adding SDS loading buffer, and the proteins were resolved on SDS-PAGE gels. Proteins were transferred onto PVDF membranes, and the tritium signal was amplified by treating membranes with EN_3_HANCE spray (PerkinElmer). Membranes were exposed to autoradiography film for at least 1 week at −80 °C.

### PTM analysis using mass spectrometry

Protein bands of interest were excised and sliced into cubes of 1 mm^3^. The gels were subjected to a conventional in-gel digestion procedure with minor modifications [38]. A schematic diagram illustrating the mass spectrometry analysis is presented in Fig. S5. As trypsin digests lysine or arginine, chymotrypsin was used to identify the methylation in these amino acids. First, 25 ng/μL of sequencing grade chymotrypsin was added to the gel slices in the reaction buffer (100 mM Tris–HCl pH 7.8, 10 mM CaCl_2_, freshly prepared). The digested peptide samples were extracted and subjected to LC–MS/MS analysis. The prepared samples were analyzed using an LTQ XL linear trap mass spectrometer (Thermo Fisher Scientific, Waltham, MA, USA) equipped with a nano-HPLC system (Eksigent, Dublin, CA, USA). Samples (5 μL) were injected and separated using an in-line, homemade reverse-phase C18 micro column, which was packed in a fused-silica nanospray tip (column dimension, 10 cm × 100 μm; particle size, 5 μm; pore size, 300 Å; Nanobaume, Wildomar, CA, USA). The peptides were eluted using the mobile phase gradient of solvent A (0.1% formic acid in water) and B (0.1% formic acid in acetonitrile) with a flow rate of 200 nL/min. The gradient started with 2% solvent B and increased to 50% by 100 min and increased by 100% after 5 min (i.e., 105 min). Nanospray ionization was carried out at 1.4 kV. Ions were first analyzed with a full-MS scan; thereafter, top seven ions with highest intensity were data-dependently selected from the full-MS scan for CID tandem MS analysis (normalized collision energy of 35 for 30 ms). The following dynamic exclusion parameters of the data-dependent scan were used: repeat count = 2, repeat duration = 30 s, list size = 300, exclusion duration = 180 s, low mass width = 0.8, and high mass width = 2.2.

### Database search

Tandem mass spectra were analyzed using SEQUEST module of Proteome Discoverer (Thermo Fisher Scientific, version 1.4.1.14). SEQUEST was set up to search UniProt human reference proteome database (UP000005640, 74601 entries, downloaded on April 28, 2020). Thereafter, 101–220 residue sequences of recombinant GST-PI3KC2α were added to the database. Chymotrypsin was used as the digestion enzyme for respective samples. SEQUEST was searched with fragment ion mass tolerance of 0.50 Da and a parent ion tolerance of 1.00 Da. Carbamidomethyl of cysteine was specified in SEQUEST as a fixed modification. Monomethyl (+ 14) or dimethyl (+ 28) of lysine or arginine, and oxidation of methionine were specified in SEQUEST as variable modifications. MS/MS based peptide and protein identifications were validated using Scaffold (version Scaffold_4.10.0, Proteome Software Inc., Portland, OR, USA). Peptide identifications were accepted if they exceeded SEQUEST thresholds; minimum charge dependent cross-correlation score of 1.8 at + 1, 2.5 at + 2, and 3.5 at + 3 or above and minimum 0.1 of DeltaCn. Proteins that contained similar peptides and could not be differentiated based on MS/MS analysis alone were grouped based on the principles of parsimony.

### Public data analysis

Cancer Cell Line Encyclopedia data were downloaded as ‘Expression Public 24Q2 data’ from the DepMap database (https://depmap.org/portal/. ; accessed on 29 July 2024).

### Immunoblotting and immunoprecipitation

As described previously [39], cells were lysed using NP-40 (10 mM Tris–HCl pH 7.4, 100 mM NaCl, 1 mM EDTA, 1 mM EGTA, 1% NP-40, 10% glycerol) or RIPA lysis buffer (50 mM Tris–HCl pH 8, 150 mM NaCl, 0.5% sodium deoxycholate, 0.1% SDS, 1% Triton X-100) supplemented with 1 × protease and phosphatase inhibitor cocktails (Roche, Basel, Switzerland). Sonication was used to lyse cells, and the obtained lysate was centrifuged at 16,000 × *g* for 10 min at 4 °C. Protein concentration of the lysate was quantified using Bradford assay (Bio-Rad, Hercules, CA, USA) in accordance with the manufacturer’s instructions.

For immunoprecipitation, after centrifugation and quantification, appropriate antibody was added to obtain lysate-adjusted concentration of 1 mg/ml; the mixture was incubated overnight at 4 °C on a rotator. Thereafter, antibody–protein complex was obtained using protein A/G Sepharose beads (Santa Cruz Biotechnology) at 4 °C by placing the mixture on a rotator for 2 h. After washing twice with lysis buffer, the complex was eluted and analyzed using SDS-PAGE. Proteins subjected to SDS-PAGE were transferred to a PVDF membrane (Millipore, Billerica, MA, USA), which was then blocked with 5% skim milk/0.1% Tween 20/TBS for at least 1 h at room temperature (RT). Then, it was incubated with primary antibody overnight at 4 °C. After washing thrice with TBS-T, the membrane was incubated with HRP-linked secondary antibody for 1 h at RT. The signal was detected using ECL western blotting substrate (Advansta, Menlo Park, CA, USA). The intensity of protein bands was analyzed using Image Studio Lite Version 5.0 (LI-COR Biotechnology, Lincoln, NE, USA).

### Microtubule co-sedimentation assay

Cells were incubated with 1 μM paclitaxel at 37 °C for 30 min and dissolved in a microtubule polymerization buffer (80 mM K-PIPES pH 6.8, 1 mM EGTA, 1 mM MgCl_2_, 0.5% NP-40, 20 mM NaF, 0.5% sodium deoxycholate, 0.1 mM PMSF, and 10 mM paclitaxel) supplemented 1 × protease inhibitor cocktail (Roche) at 37 °C for 5 min in the dark. The microtubule fraction was separated by centrifugation at 16,000 × g for 15 min at 30 °C. The microtubule fraction (pellet, insoluble) and the supernatant (soluble) were collected separately. The pellet was washed with microtubule polymerization buffer without detergent and resuspended with the same buffer in an equal volume of supernatant. Both fractions were loaded for SDS‐PAGE, followed by western blotting.

### Immunofluorescence and confocal microscopy

Cells were plated on coverslips for 24 h and then incubated with CARM1 or PI3KC2α siRNA for 72 h. The cells were fixed for 15 min in 4% paraformaldehyde (PFA), washed thrice with PBS, and permeabilized using 0.5% Triton X-100 for 15 min at room temperature. Thereafter, the cells were again washed thrice with PBS and incubated with primary antibody overnight at 4 °C, followed by secondary antibody conjugated with FITC and Alexa Fluor 594 (Bethyl Laboratories, A90-116D4, A90-138D2, A120-101D4, and A120-101F). Staining was visualized using Zeiss LSM 710 Confocal Microscopy (Carl Zeiss, Oberkochen, Germany), and images analysis was performed using ZEN or image J software.

### Quantitative real-time PCR

Total cellular RNA was extracted using TRIsure (Bioline, London, UK), and cDNA was synthesized using the SensiFAST cDNA synthesis kit (Bioline). To measure pre-mRNA levels, random hexamer primers were used instead of oligo(dT)_18_ primers when synthetizing cDNA. Levels of mRNA were analyzed via quantitative real-time PCR using SensiFAST SYBR No-ROX kit (Bioline) and Eco Real-Time PCR system (Illumina, San Diego, CA, USA). Reaction parameters were as follows: cDNA synthesis at 40 °C for 60 min, transcriptase inactivation at 95 °C for 5 min, and PCR cycling at 95 °C for 10 s, 58 °C for 20 s, and 72 °C for 20 s (40 cycles). To measure pre-mRNA levels, random hexamer primers were used instead of oligo(dT)_18_ primers when synthesizing cDNA.

**Table:** 

**Primer sets**
*ACTB*(human) F: 5′-ACCTTCTACAATGAGCTGCG-3′ and R: 5′-CCTGGATAGCAACGTACATGG-3′
*Actb *(mouse) F: 5′-ACCTTCTACAATGAGCTGCG-3′ and R: 5′-CTGGATGGCTACGTACATGG-3′
*CARM1 *(human) F: 5′-TTTAAGTGCTCAGTGTCCCG-3′ and R: 5′-GGCAGGTTTTCAGGATGTTG-3′
*Carm1 *(mouse) F: 5′-GTTTTCAAGTGCTCGGTGTC-3′ and R: 5′-CGACAGGTTTTCAGGATGTTG-3′
*PIK3C2A* (human/mouse) F: 5′-GGAAAGCAAGGACTGATTTGG-3′ and R: 5′-AGGGATTTTCCATTCACCTTTC-3′
*STMN1 *(human) F: 5′-AGCCCTCGGTCAAAAGAATC-3′ and R: 5′-TTCAAGACCTCAGCTTCATGG-3′
*TUBA1A* (human) F: 5′-TTGTAGACTTGGAACCCACAG-3′ and R: 5′-ATCTCCTTGCCAATGGTGTAG-3′
*Tuba1a* (mouse) F: 5′-CAGTGTTCGTAGACCTGGAAC-3′ and R: 5′-ATCTCCTTGCCAATGGTGTAG-3′
*TUBB *(human) F: 5′-GAAGCCACAGGTGGCAAATA-3′ and R: 5′-CGTACCACATCCAGGACAGA-3′
*TUBA1A* pre-mRNA F: 5′-GCAGCATTTGTAGCAGGTGA-3′ and R: 5′-GCATTGCCAATCTGGACAC-3′
*TUBB* pre-mRNA F: 5′-CTGGACCGCATCTCTGTGTA-3′ and R: 5′-GGTTCACGAAAGGGACAAAA-3′
*GAPDH* pre-mRNA F: 5′-GGGAGGTAGAGGGGTGATGT-3′ and R: 5′-GAGGCAGGGATGATGTTCTG-3′

### Cell synchronization

To arrest cells at the G_1_/S stage, MCF7 cells were seeded at 30% confluence and were maintained in growth media added with 2 mM thymidine (Sigma-Aldrich) for 18 h. The cells were washed with PBS and placed in the fresh growth media for 9 h. Thereafter, 2 mM thymidine was re-added to the media and cells were incubated with it for 15 h. To arrest cells in the G_2_/M phase, cells were allowed to react with 100 nM BI2536 (Sigma-Aldrich) for 24 h or 100 ng/ml nocodazole for 12 h. For metaphase arrest, cells synchronized in the G_2_/M phase were released in fresh media containing 20 μM MG132 (Sigma-Aldrich) for 2 h.

### Flow cytometry analysis

To analyze cell cycle, cells were harvested using trypsin, washed with PBS, and then fixed using 70% ethanol for at least 1 h on ice. After washing the fixed cells with PBS, they were incubated with 0.2 mg/ml RNase A at 37 °C for 1 h. The cells were then washed with PBS and stained with 10 μg/ml propidium iodide (BD Biosciences). Data measured by FACSCalibur flow cytometer (BD Biosciences) were analyzed using FlowJo software.

### EdU incorporation assay

Cell proliferation rates were measured using EdU proliferation kit (Abcam, Cambridge, UK), according to the manufacturer’s instructions. Briefly, cells were seeded, pulse-labeled with 20 μM EdU for 2–3 h, and harvested as described above. Harvested cells were washed with PBS and 3% BSA and fixed with 4% formaldehyde. After 15 min of incubation in dark, the cells were washed and permeabilized. Thereafter, EdU reaction mixture, including CuSO_4_, iFluor 488 azide, and sodium ascorbate, was prepared and added to the cells. The samples were incubated for 30 min at room temperature in the dark, washed, and transferred to new tubes on ice.

### Cell counting, colony formation and MTT assays

Cell growth and viability was determined via cell counting, colony forming, and MTT assay. In cell counting, cells were seeded in 12-well plates and knocked down using CARM1 or PI3KC2α siRNA. After 72 h, the cells were replated and treated with paclitaxel or vincristine. After harvesting using trypsin, cells were washed with PBS and counted using Z2 Coulter Counter (Beckman Coulter, Brea, CA, USA). The diameter range of counted cells was between 10 and 30 μm. Growth inhibition was calculated as a percentage relative to the control group. A decrease in cell counts after drug treatment compared to the initial seeding counts indicates cell death (cytotoxicity). In case of such reduction, this was expressed as a percentage relative to the initial seeding counts.

In colony forming assay, cells were transfected with CARM1 or PI3KC2α siRNA for 72 h and then re-seeded with fresh complete media in 6-well plates at a density of 1,000 cells/well. After 5 d of culture, the colonies were fixed with 4% PFA and stained using 0.05% crystal violet. The stained cells were washed with deionized water and then dried. After dissolving the stained crystal violet in a 30% acetic acid solution, the absorbance at 540 nm was measured using an Epoch Microplate Spectrophotometer (BioTek, Winooski, VT, USA).

In MTT assay, cells seeded in 96-well plates were treated with various concentration of vincristine or paclitaxel for 48 h, and 0.5 mg/ml MTT was added to each well. After incubation for 2 h, media was discarded and 120 μL of DMSO was added to each well. The absorbance was measured at 590 nm using Epoch Microplate Spectrophotometer.

### Statistical analysis

All statistical analyses were performed in GraphPad Prism software. The data from independent experiments were included and presented as mean ± standard deviation (*n* ≥ 3). Data from two groups were compared using unpaired t-test for independent samples, and *p*-value < 0.05 was considered statistically significant. *: *p* < 0.05, **: *p* < 0.01, and ***: *p* < 0.001.

## Supplementary Information

Supplementary Material 1

## Data Availability

No datasets were generated or analysed during the current study.
