# Supplementary material for: CARM1 regulates tubulin autoregulation through PI3KC2α R175 methylation
Source: Cell Commun Signal. 2025 Mar 5;23:120. doi: 10.1186/s12964-025-02124-z (PMC11884010; doi:10.1186/s12964-025-02124-z)
Supplement: Supplementary file 1 — Supplementary Material 1 [file 12964_2025_2124_MOESM1_ESM.docx]

**[Supplementary Information]**

**CARM1 regulates tubulin autoregulation through PI3KC2α R175 methylation**

Yena Cho^1,2^, Jee Won Hwang^2^, Mark T Bedford^3^, Dae-Geun Song^4,5^, Su-Nam Kim^4,5^, and Yong Kee Kim^1,2,*^

^1^Muscle Physiome Research Center and Research Institute of Pharmaceutical Sciences, Sookmyung Women’s University, Seoul 04310, Republic of Korea

^2^College of Pharmacy, Sookmyung Women’s University, Seoul 04310, Republic of Korea

^3^Department of Epigenetics and Molecular Carcinogenesis, The University of Texas MD Anderson Cancer Center, Houston, TX, 77030, USA

^4^Natural Products Research Institute, KIST Gangneung, Gangneung 25451, Republic of Korea

^5^Division of Natural Product Applied Science, University of Science and Technology KIST School, Seoul 02792, Republic of Korea

***Correspondence to:**

Yong Kee Kim, Ph.D., E-mail: [yksnbk@sookmyung.ac.kr](mailto:yksnbk@sookmyung.ac.kr), Tel: +82-2-2077-7688, Fax: +82-2-710-9871


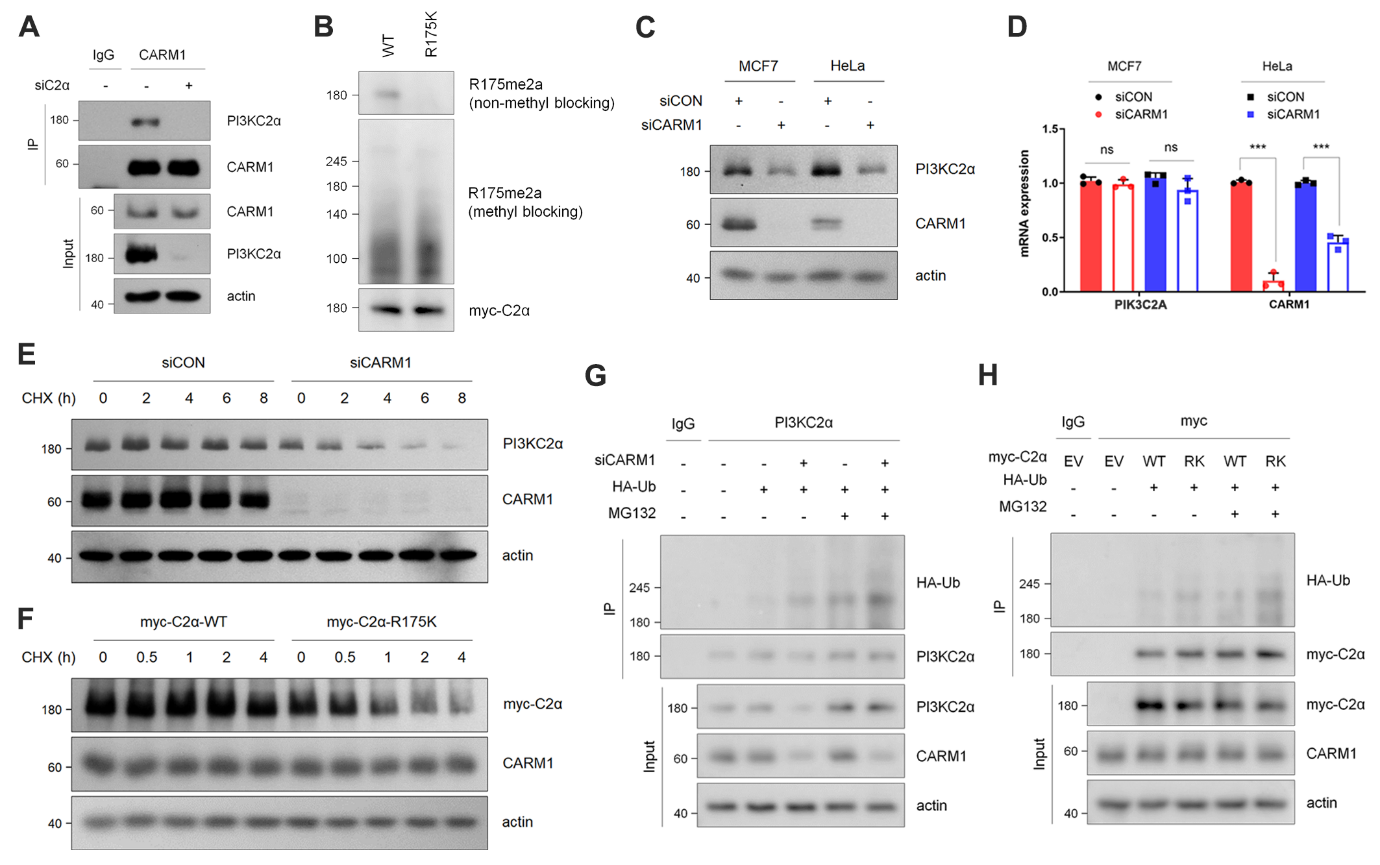


**S1 Fig. CARM1 methylates and stabilizes PI3KC2α at R175 residue.**

(**A**) Physical interactions between CARM1 and PI3KC2α were detected by immunoprecipitating CARM1 in PI3KC2α-knockdown cells. (**B**) Immunoblot analysis after blocking with non-methyl or methyl peptide to validate anti-PI3KC2α-R175me2a antibody. (**C** and **D**) The levels of PI3KC2α protein (C) and mRNA (D) in either MCF7 or HeLa cells treated with CARM1 siRNA for 72 h. Data are indicated as means ± standard deviations (n = 3). (**E**) Levels of PI3KC2α were measured in MCF7 cells transfected with control or CARM1 siRNA for 72 h and treated with cycloheximide (CHX, 50 μg/ml) for the indicated time. (**F**) Expression of myc-PI3KC2α was measured after treatment of CHX in cells in which either WT or R175K-mutant of myc-PI3KC2α was overexpressed. (**G** and **H**) Ubiquitination assays in MCF7 cells transfected with control or CARM1 siRNA (G) and myc-PI3KC2α WT or R175K (H) for 72 h. HA-Ub was co-transfected and treated with 10 μM MG132 for 6 h. Ubiquitinated PI3KC2α was probed with an anti-HA antibody.


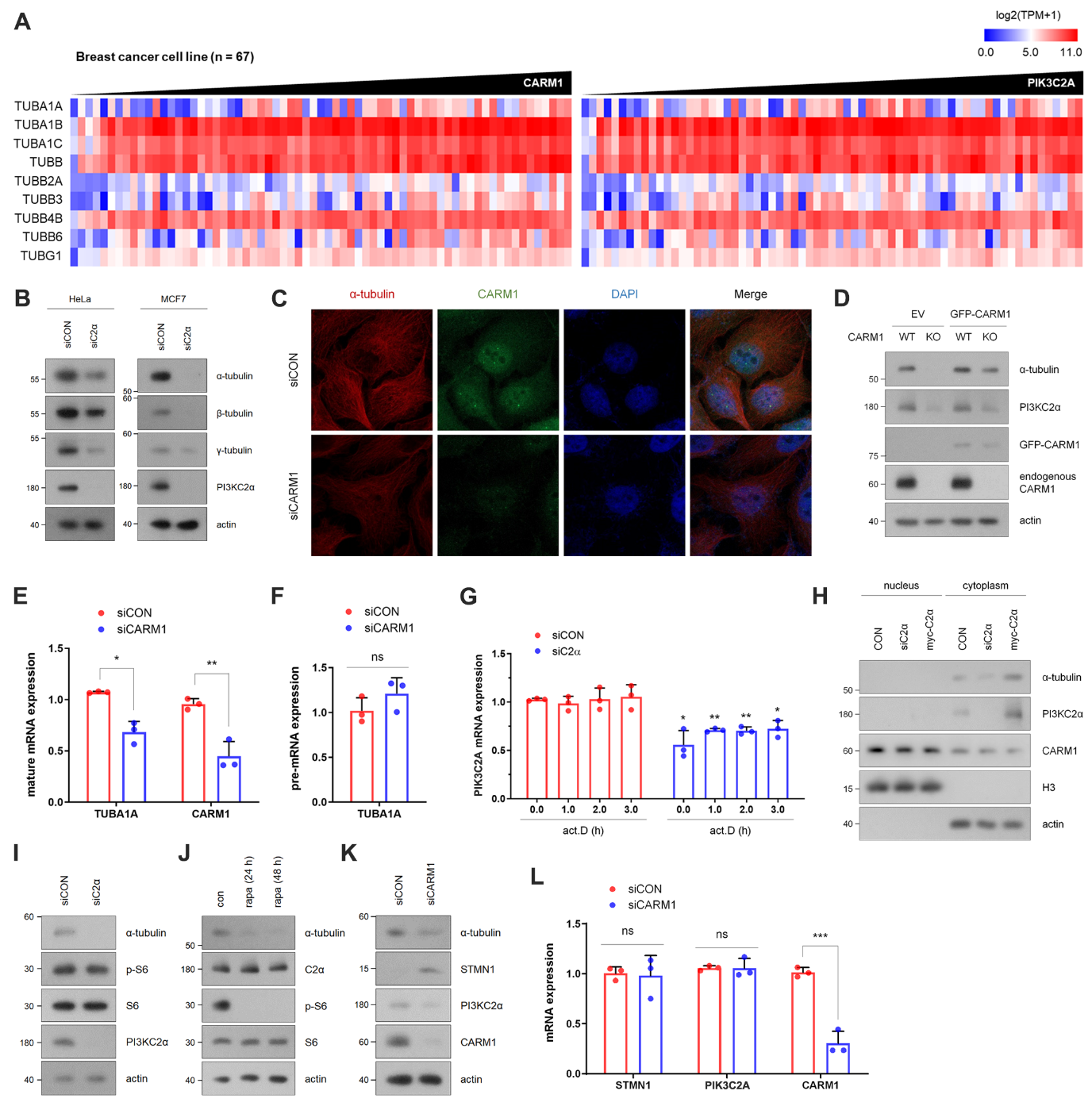


**S2 Fig. CARM1–PI3KC2α axis determines intracellular tubulin levels.**

(**A**) Heat map of log2(TPM+1) values of tubulin in 67 breast cancer cell lines, derived from DepMap Expression Public 24Q2 data. (**B**) Cell lysates from HeLa or MCF7 cells after knockdown of PI3KC2α were analyzed using immunoblotting to detect α-, β-, and γ-tubulin levels. (**C**) Representative confocal images showing α-tubulin (red) and CARM1 (green) levels. DAPI (blue) was used to stain cell nuclei. (**D**) CARM1 WT or KO MEF cells were transfected with GFP-CARM1 vector and analyzed using immunoblotting. (**E** and **F**) Levels of mature mRNA (E) and pre-mRNA (F) of *TUBA1A* were measured in CARM1-knockdown cells. Error bar indicates standard deviation (n = 3). (**G**) PI3KC2α mRNA levels were measured at different time points (1, 2, and 3 h) after treatment with actinomycin D (act.D) in cells transfected with PI3KC2α siRNA for 60 h. Error bar indicates standard deviation (n = 3). (**H**) Nuclear and cytoplasmic fractions that were prepared using MCF7 cells after knockdown or overexpression of PI3KC2α were analyzed using immunoblotting. Histone H3 and actin were used as nuclear and cytoplasmic markers, respectively. (**I** and **J**) To measure the effect on α-tubulin translation, S6 phosphorylation levels were measured after treatment with PI3KC2α siRNA for 72 h (I) or 50 nM rapamycin for 24 or 48 h (J). (**K** and **L**) Levels of STMN1 protein (K) and mRNA (L) were measured in CARM1-knockdown cells. Data are indicated as means ± standard deviations (n = 3).

**
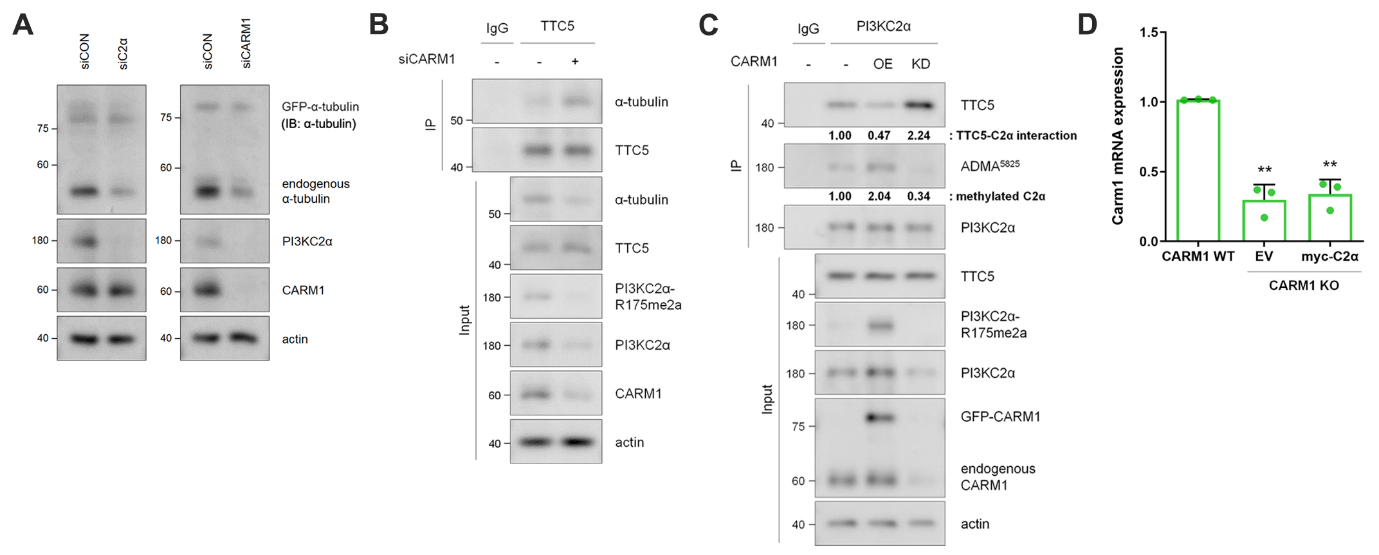
**

**S3 Fig. PI3KC2α regulates tubulin autoregulation by sequestering TTC5.**

(**A**) Cell lysates from HeLa cells stably expressing GFP-α-tubulin were immunoblotted to measure the levels of GFP- and endogenous-α-tubulin after transfection with either PI3KC2α or CARM1 siRNA for 72 h. (**B**) Physical interactions of α-tubulin with TTC5 were measured using co-IP experiments after transfecting the cells with CARM1 siRNA. (**C**) Immunoblot analysis was used to measure the methylation level of PI3KC2α and its interaction with TTC5 after immunoprecipitating PI3KC2α in cells that overexpressing or knocked down CARM1. (**D**) Myc-PI3KC2α vector was transfected into CARM1 KO MEF cells and the mRNA levels of *Carm1* in each group were measured using quantitative real-time PCR. Data are presented as means ± standard deviations (n = 3).


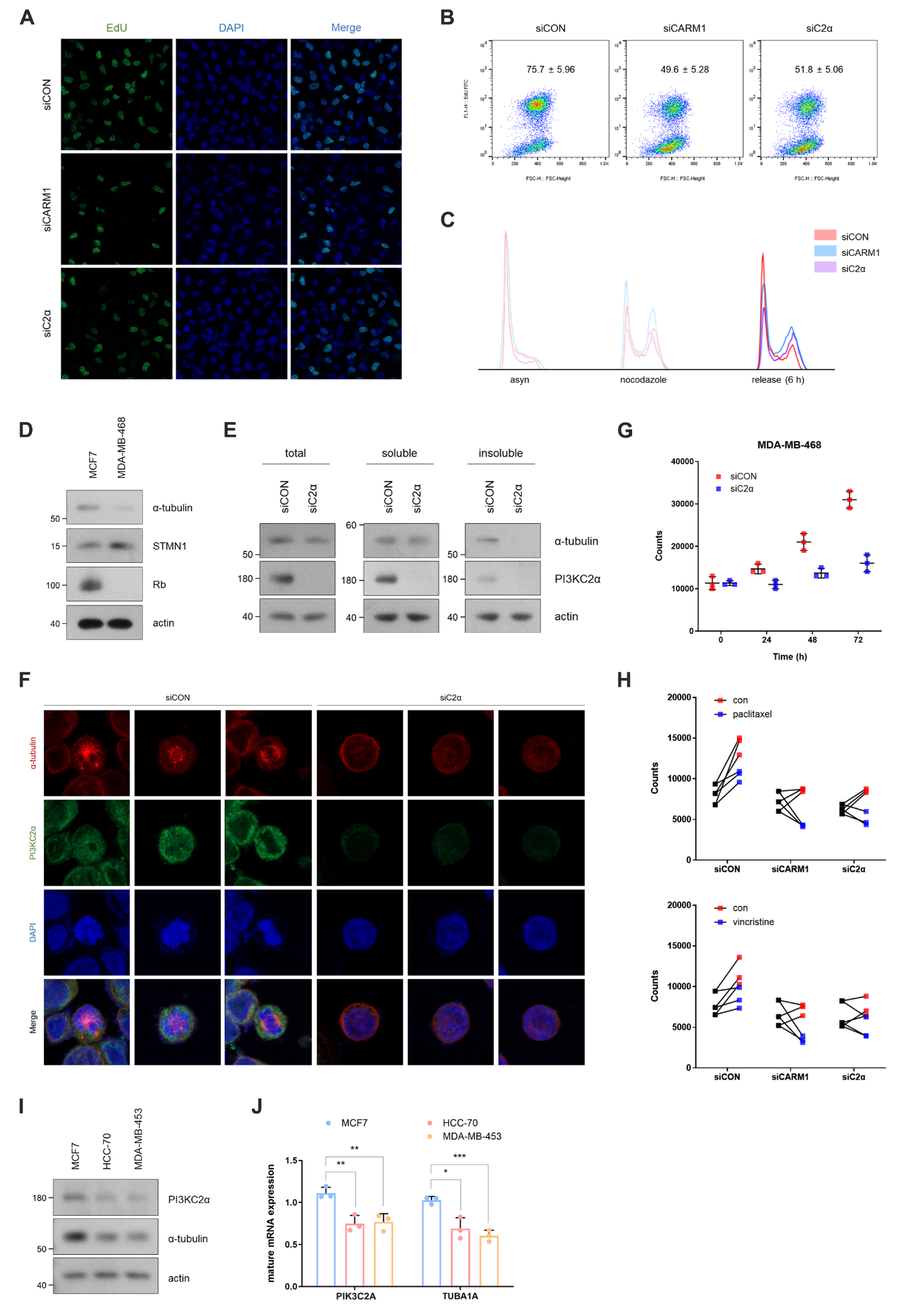


**S4 Fig. Disruption of the CARM1–PI3KC2α axis enhances the cytotoxic effects of microtubule-targeting agents.**

(**A** and **B**) Cells were transfected with siRNA that targets CARM1 or PI3KC2α and incubated with 20 μM EdU for 3 h. EdU signals were detected using confocal microscopy (A) and flow cytometry (B). (**C**) After synchronizing CARM1- or PI3KC2α-knockdown cells in prometaphase with nocodazole, the cells were released by incubation with fresh media for 6 h. The cell cycle was analyzed *via* flow cytometry and visualized using FlowJo software. (**D**) Immunoblot results showing the levels of α-tubulin, STMN1, and Rb in MCF7 or MDA-MB-468 cells. (**E**) Levels of α-tubulin were determined using immunoblotting in total lysates and soluble/insoluble fractions after knockdown of PI3KC2α in MDA-MB-468 cells. (**F**) Immunostaining with α-tubulin (red) and PI3KC2α (green) reveals abnormal mitosis in PI3KC2α-knockdown cells. (**G**) MDA-MB-468 cells were transfected with PI3KC2α siRNA for 72 h, and cell growth was measured *via* cell counting using a coulter counter after incubation with fresh media for the indicated time. Data are expressed as means ± standard deviations (n = 3). (**H**) MCF7 cells were transfected with siRNA targeting CARM1 or PI3KC2α. The cells were then replated with fresh media and treated with either 3 nM paclitaxel or vincristine. Cell counting was performed after 48 h of incubation. The individual counts were plotted (n = 3). (**I** and **J**) PI3KC2α and α-tubulin levels were measured by immunoblotting (H) and quantitative real-time PCR (I) in MCF7, HCC-70, or MDA-MB-453 cells. Data are indicated as means ± standard deviations (n = 3).


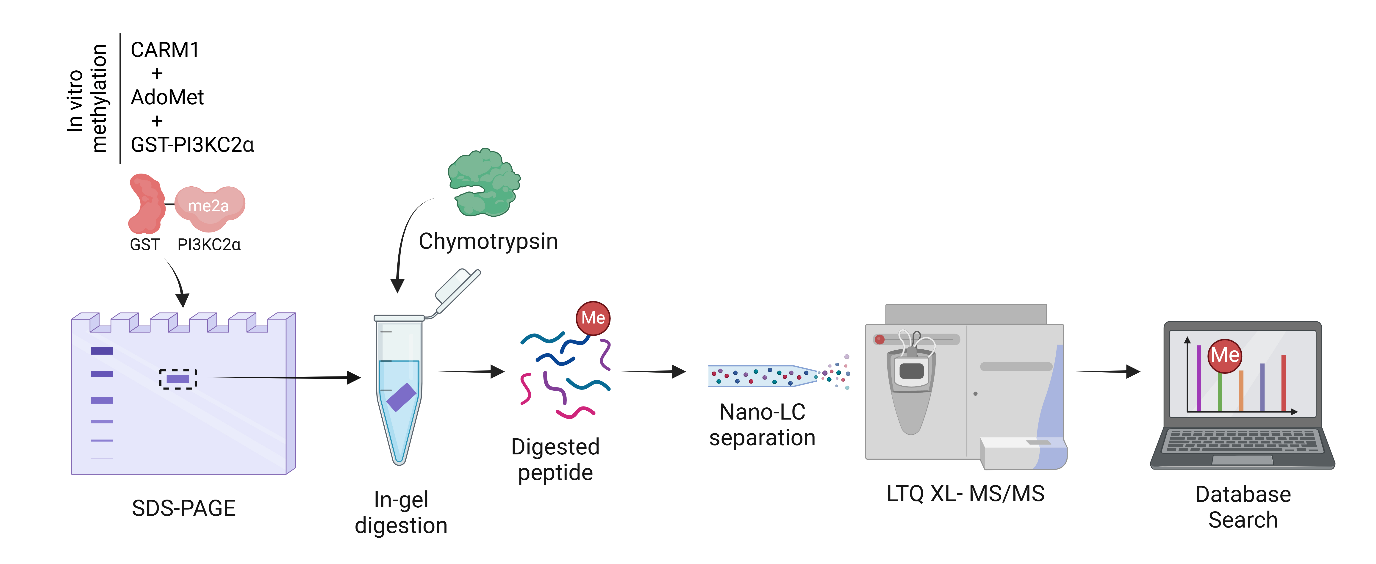


**S5 Fig. A schematic diagram to illustrate the mass spectrometry analysis**

**[Uncropped Gels and Blots]**

**Fig 1.**

**
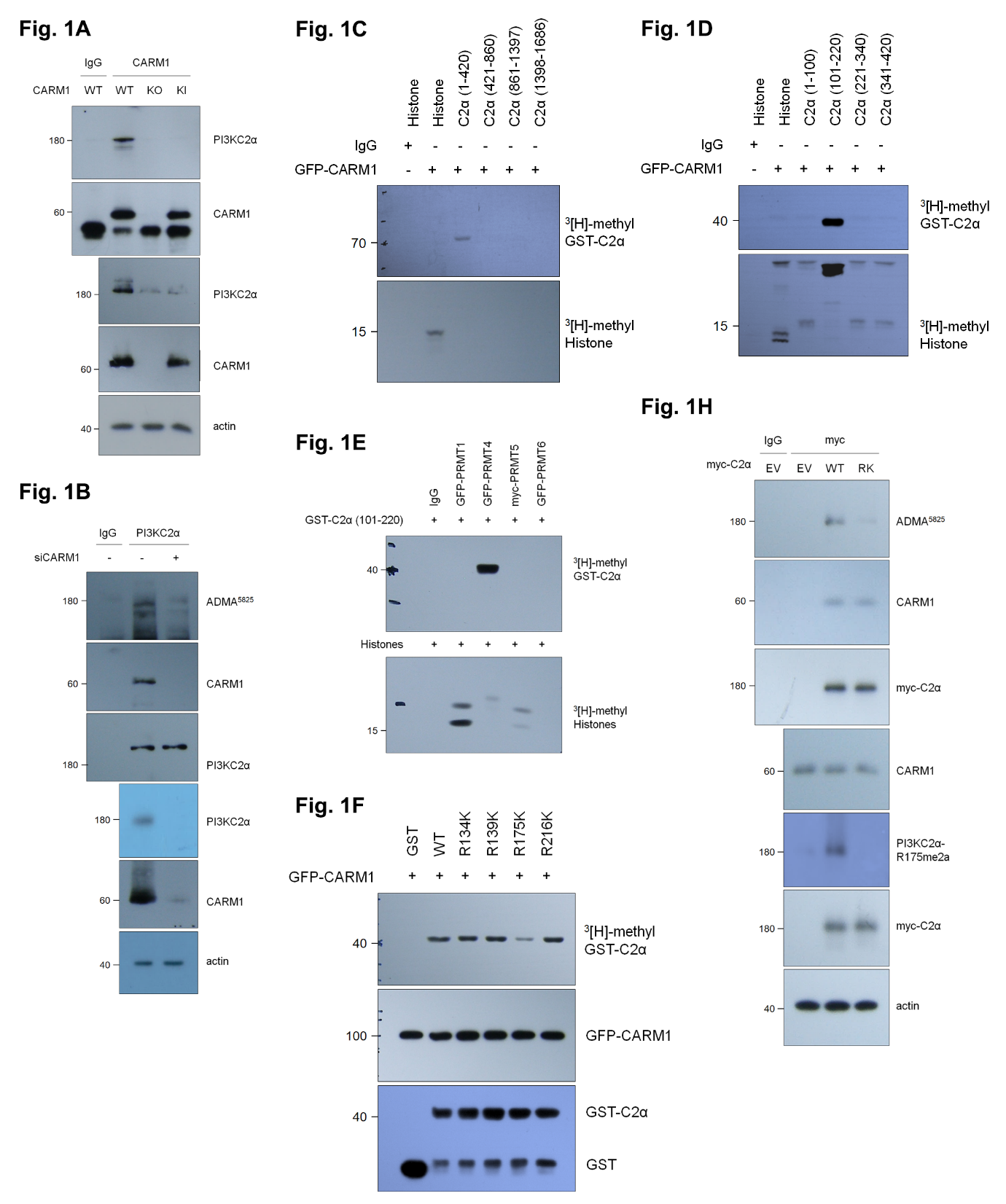
**

**S1 Fig.**

**
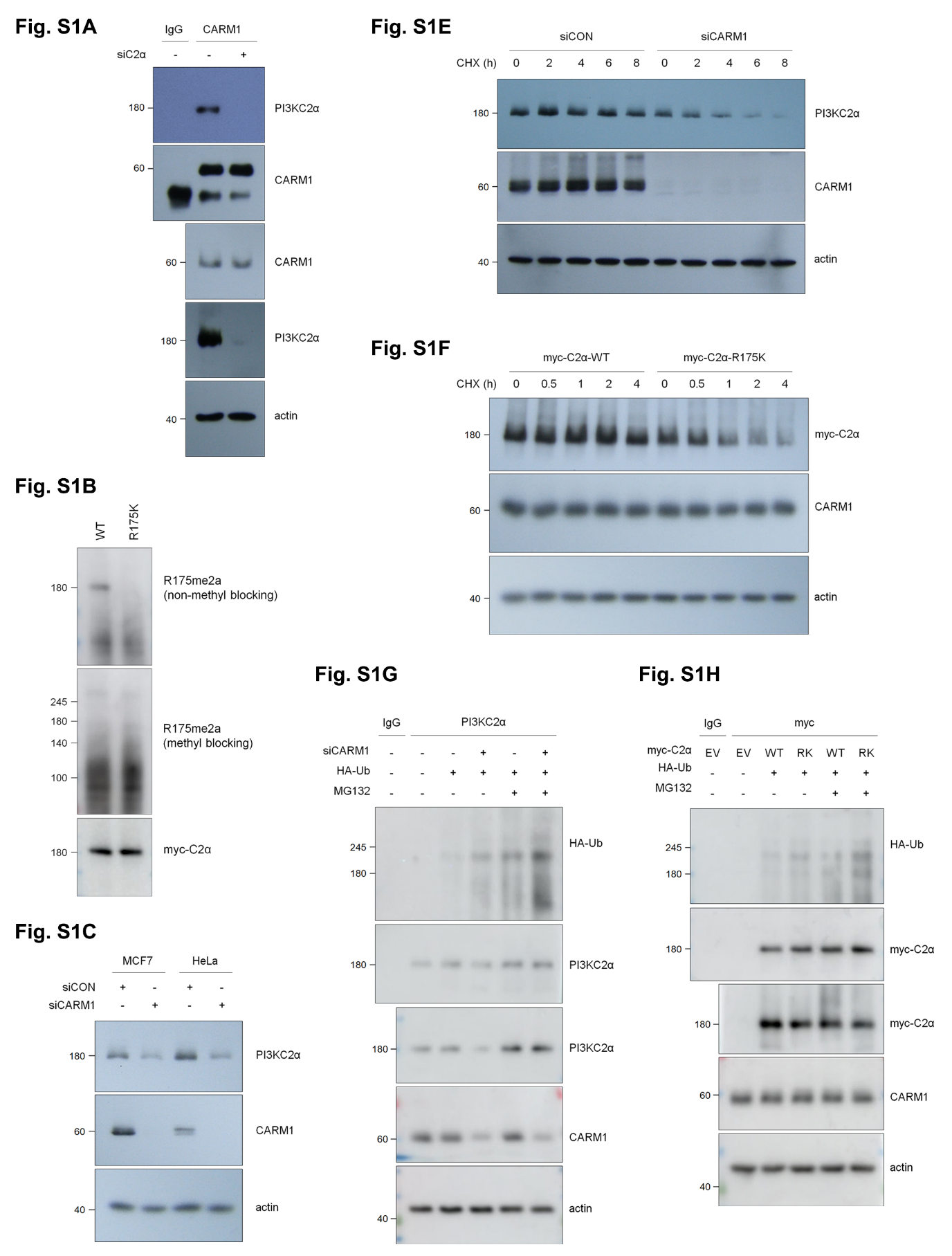
**

**Fig 2.**

**
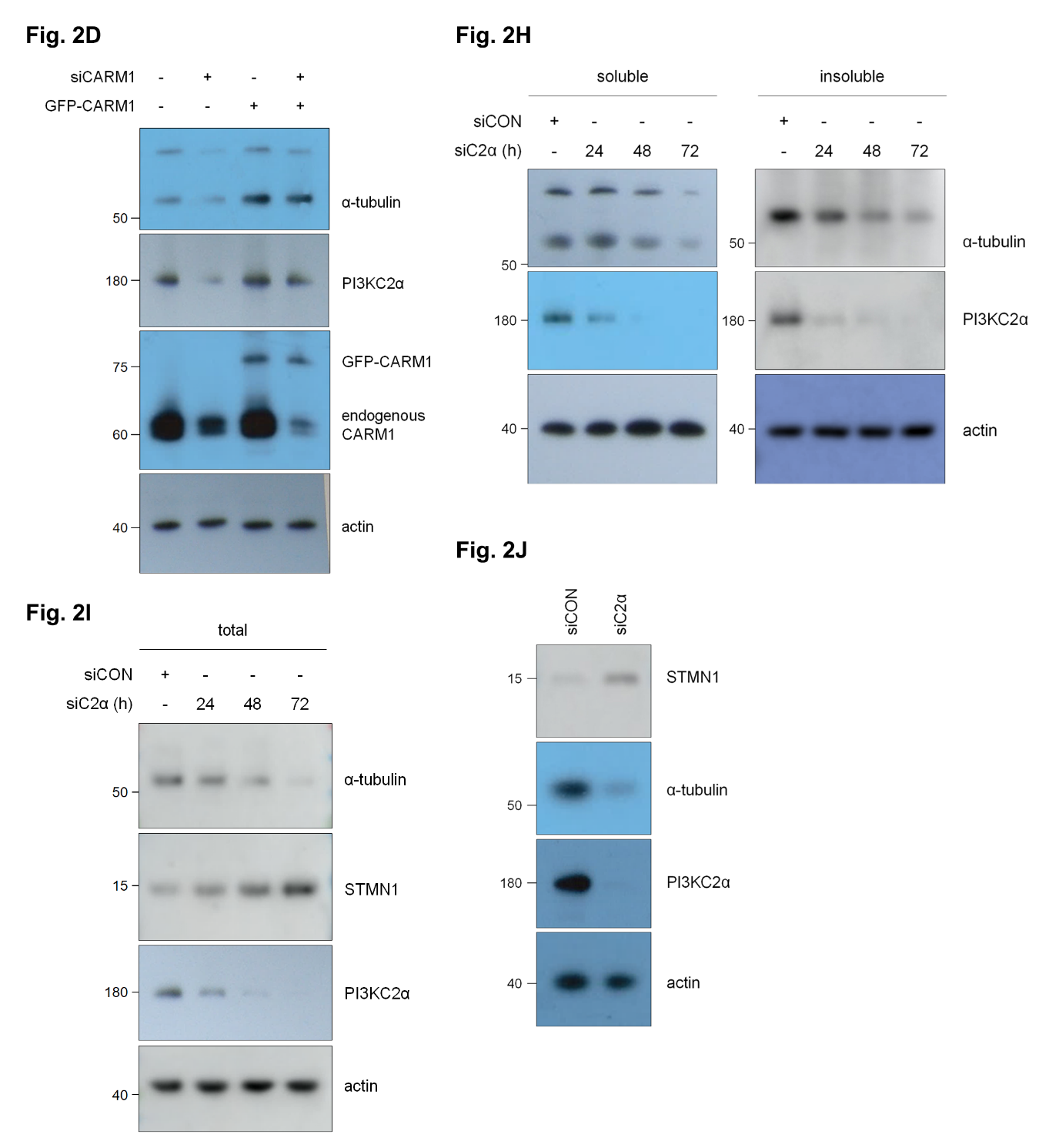
**

**S2 Fig.**

**
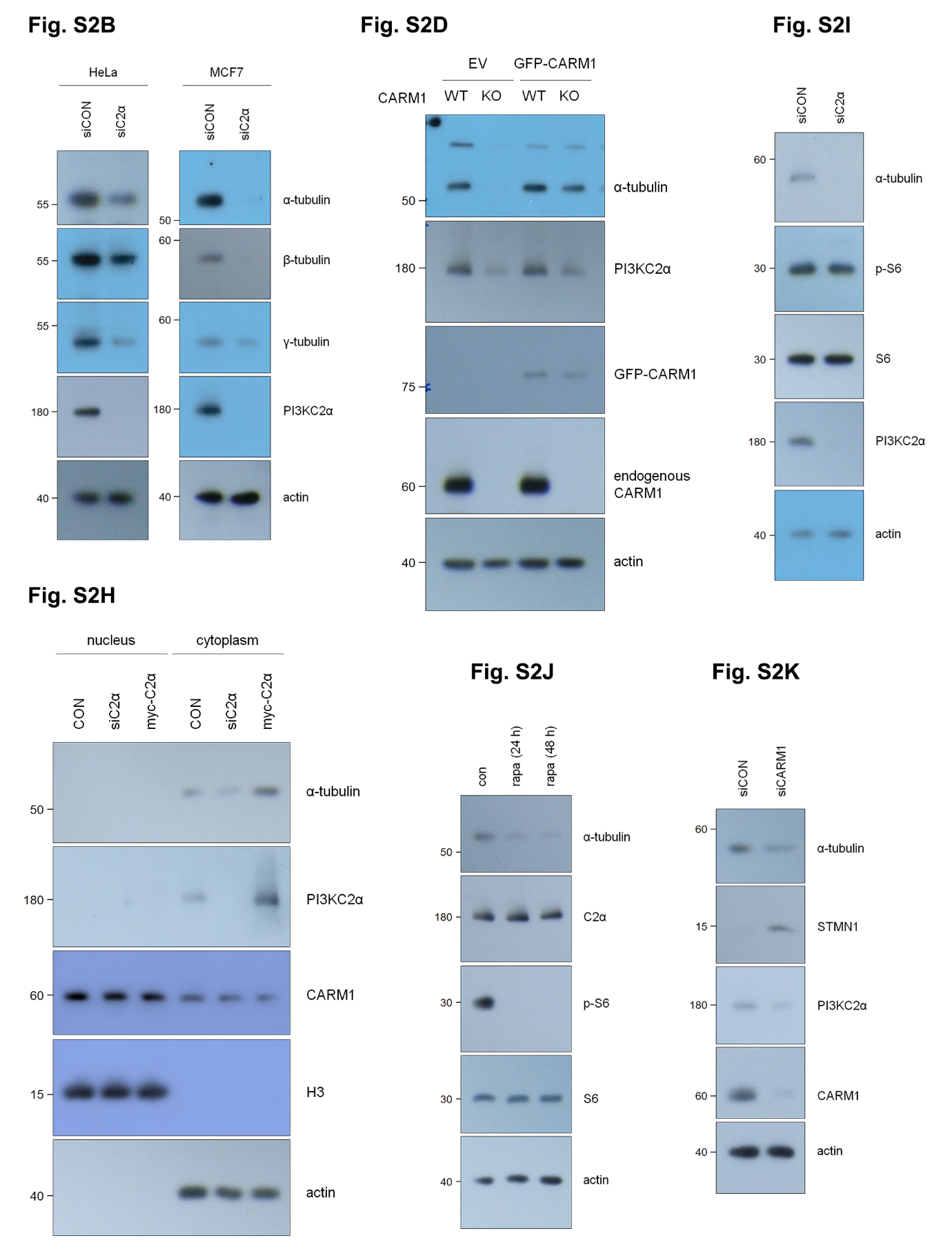
**

**Fig 3.**

**
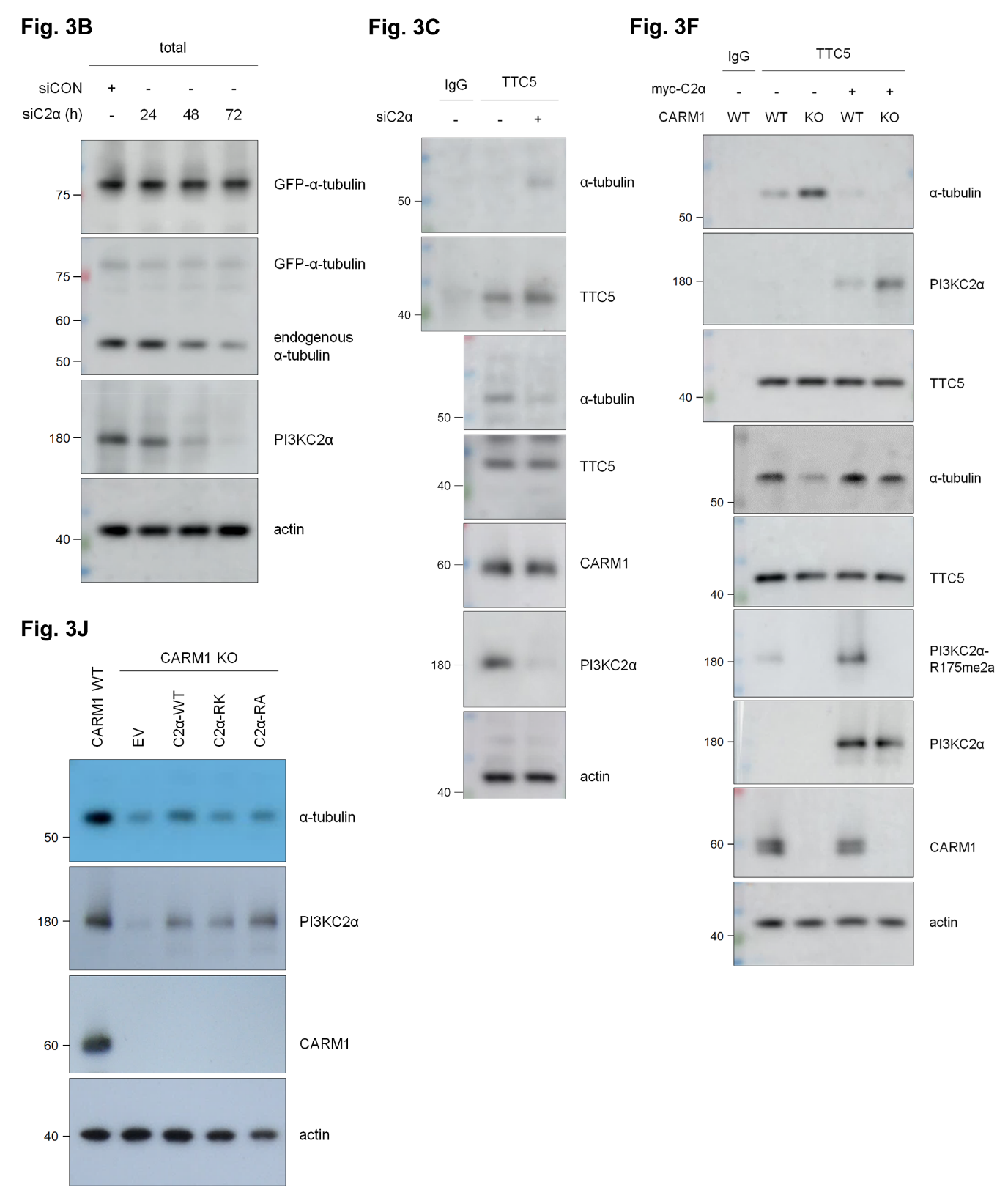
**

**S3 Fig.**

**
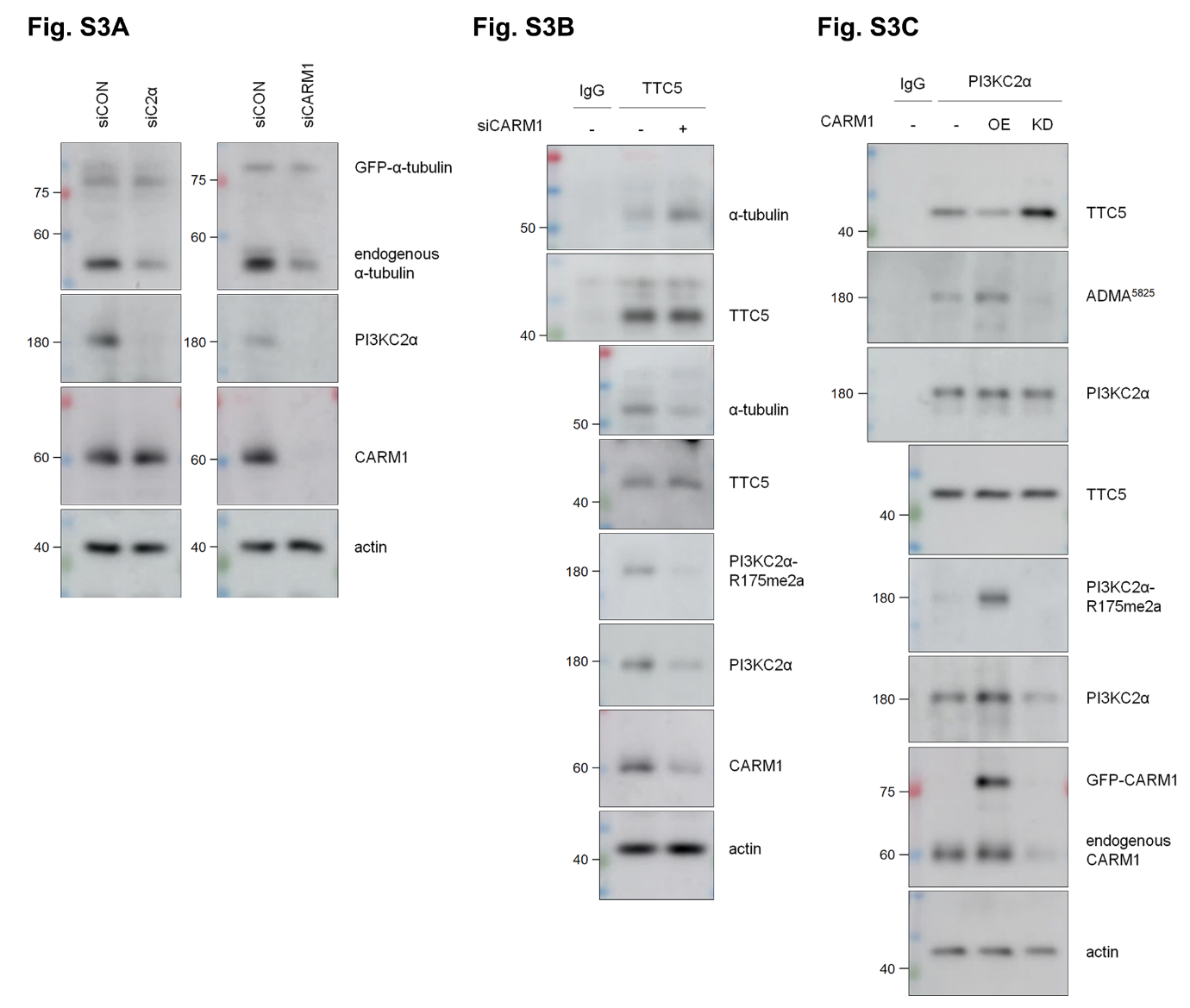
**

**Fig 4. and S4 Fig.**

**
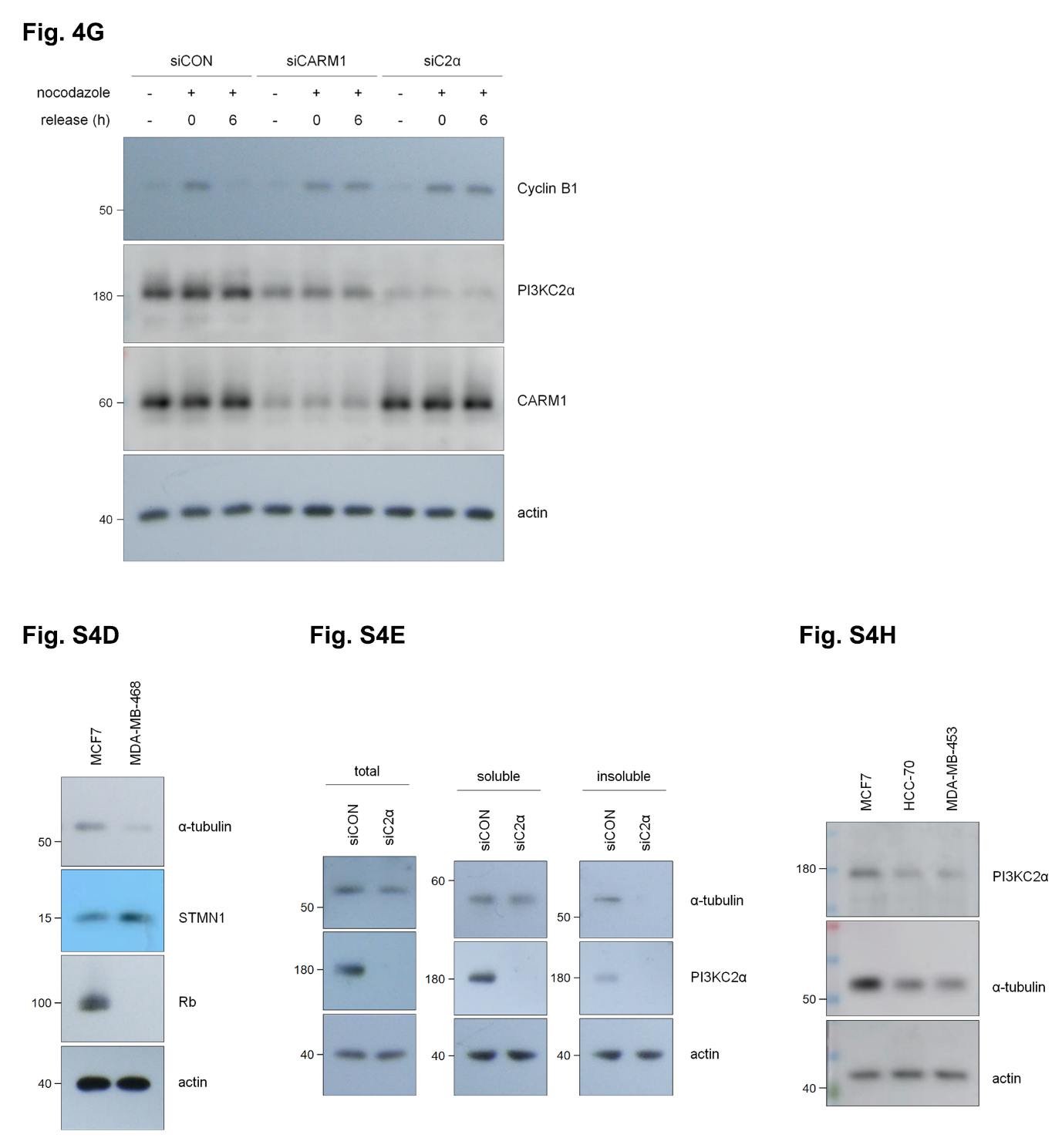
**
